# The impact of the cardiovascular component and somatic mutations on ageing

**DOI:** 10.1111/acel.13957

**Published:** 2023-08-22

**Authors:** Daniel Garger, Martin Meinel, Tamina Dietl, Christina Hillig, Natalie Garzorz‐Stark, Kilian Eyerich, Martin Hrabě de Angelis, Stefanie Eyerich, Michael P. Menden

**Affiliations:** ^1^ Computational Health Center, Helmholtz Munich Neuherberg Germany; ^2^ Faculty of Biology Ludwig Maximilian University Martinsried Germany; ^3^ Department of Dermatology and Allergy Technical University of Munich Munich Germany; ^4^ Department of Mathematics Technical University of Munich Munich Germany; ^5^ Division of Dermatology and Venereology, Department of Medicine Solna, and Center for molecular medicine Karolinska Institutet Stockholm Sweden; ^6^ Department of Dermatology and Venerology, Medical School University of Freiburg Freiburg Germany; ^7^ Institute of Experimental Genetics Helmholtz Munich Neuherberg Germany; ^8^ Chair of Experimental Genetics, TUM School of Life Sciences Technical University Munich Freising Germany; ^9^ German Center for Diabetes Research (DZD) Neuherberg Germany; ^10^ Center for Allergy and Environment (ZAUM) Technical University Munich Munich Germany; ^11^ Institute for Allergy Research Helmholtz Munich, Neuherberg Neuherberg Germany; ^12^ Department of Biochemistry and Pharmacology University of Melbourne Parkville Victoria Australia

**Keywords:** aging, animals, biology, biostatistics, female, humans, litter size, longevity, male, mammals, mutation, phenotype

## Abstract

Mechanistic insight into ageing may empower prolonging the lifespan of humans; however, a complete understanding of this process is still lacking despite a plethora of ageing theories. In order to address this, we investigated the association of lifespan with eight phenotypic traits, that is, litter size, body mass, female and male sexual maturity, somatic mutation, heart, respiratory, and metabolic rate. In support of the somatic mutation theory, we analysed 15 mammalian species and their whole‐genome sequencing deriving somatic mutation rate, which displayed the strongest negative correlation with lifespan. All remaining phenotypic traits showed almost equivalent strong associations across this mammalian cohort, however, resting heart rate explained additional variance in lifespan. Integrating somatic mutation and resting heart rate boosted the prediction of lifespan, thus highlighting that resting heart rate may either directly influence lifespan, or represents an epiphenomenon for additional lower‐level mechanisms, for example, metabolic rate, that are associated with lifespan.

## Graphical abstract

Lifespan prediction of mammalian species using phenotypic traits. Somatic mutation rate proved to be the strongest sole predictor among all traits. In addition, a model leveraging somatic mutation rate and resting heart rate significantly improved the prediction of lifespan.

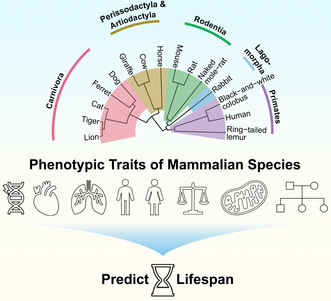

**Abbreviations**
ATP: Adenosine triphosphate
BMR: Basal metabolic rate
DNA: Deoxyribonucleic acid
FDR: False discovery rate
HRV: Heart rate variability
OLS: Ordinary least squares
PCA: Principal Component Analysis
PGLS: Phylogenetic generalised least squares
RHR: Resting heart rate
RNA: Ribonucleic acid
ROS: Reactive Oxygen Species

## 1 INTRODUCTION

Understanding processes that drive ageing and determine the longevity of organisms, has been a subject of intensive research in the past 100 years (Table 1) (Robin Harris & Korolchuk, 2019; Harley, 1991; Harman, 1956; Kanungo, 1975; Kirkwood, 1977; López‐Otín et al., 2013; Morley, 1995; Orgel, 1963; Rubner, 1908; Timiras, 2007; Vijg, 2000; Weinert & Timiras, 2003; Williams, 1957). Gaining mechanistic insight into ageing could provide us with the possibility to extend the lifespan of humans (Zhao et al., 2021). The definition of ageing is challenging as different types of ages are known (Balcombe & Sinclair, 2001): chronological age, which is defined as the time passed since birth; biological age, which is associated with the presence or absence of pathological processes; sociological age, which reflects the expected role of an individual within the society. In the last decades, the field agreed on a more general definition, namely that ageing represents a progressive accumulation of changes in the cells and organs with advancing age that lead to an increased risk of disease and death through a decline in function (Balcombe & Sinclair, 2001; Harman, 2003).

**TABLE 1:** Theories of ageing. Shows the common theories of ageing split by molecular, cellular and evolutionary level.

Theory	Description of theory
Molecular Level	
Somatic mutation (Morley, 1995; Vijg, 2000)	Accumulating mutations in the somatic DNA over time caused by endogenous or environmental factors cause functional decline
Gene regulation (Kanungo, 1975)	Changes in the expression of genes that regulate the development of the organism also affect its lifespan
Error catastrophe (Orgel, 1963)	Errors in the proteins involved in processing genetic information (DNA replication, RNA translation) contribute to an increase in the level of abnormal proteins, leading to functional decline
Cellular level	
Cellular senescence (Harley, 1991; Timiras, 2007)	Cells enter cellular senescence and present a decline in function. Causes for this can be replicative senescence (by telomere loss) or stress induced senescence
Free radical theory (Harman, 1956)	Reactive oxygen species (ROS) created by the respiratory enzymes of a cell cause ageing by damaging its macromolecules (DNA, RNA, proteins, and lipids)
Mitochondrial dysfunction (López‐Otín et al., 2013)	Dysfunctional mitochondria generate increased levels of ROS and have reduced efficiency in ATP production, thereby contributing to ageing
System level	
Rate of living (Pearl, 1928; Rubner, 1908)	Lifetime energy expenditure per gram tissue is constant among species, therefore the is an inverse relationship between metabolism and lifespan
Neuroendocrine (López‐Otín et al., 2013; Proshkina et al., 2020; Weinert & Timiras, 2003)	Changes in neuroendocrine control of homeostasis lead to a decreased ability to survive stress
Immunologic (Robin Harris & Korolchuk, 2019; Weinert & Timiras, 2003)	A dysregulation of the immune system occurs with ageing, giving rise to cancer, infectious and autoimmune diseases
Evolutionary level	
Disposable soma (Kirkwood, 1977)	Selection favours the maintenance of somatic cells until reproductive age, after that the soma is disposal
Antagonistic pleiotropy (Williams, 1957)	Genes selected by evolution for their beneficial effects in early life may have unselected unfavourable effects later

Numerous theories of ageing have been proposed trying to explain lifespan as a result of various factors on a molecular, cellular, system or evolutionary level (Marchionni et al., 2020; Proshkina et al., 2020; Weinert & Timiras, 2003). Most of them aim to trace back the cause of ageing to a single factor (Table 1). In recent decades, this view has changed as ageing is now considered a highly complex multifactorial process, that is, independently identified processes of ageing are assumed to jointly orchestrate the normal process of ageing (Kowald & Kirkwood, 1996; Weinert & Timiras, 2003). In particular, a large number of genes and proteins have been identified, which are associated with ageing and lifespan (López‐Otín et al., 2013; Proshkina et al., 2020; Weinert & Timiras, 2003). Nevertheless, we are still lacking a universal theory of ageing which remains an actively and intensively investigated field of science.

A recent publication by Cagan et al. has provided support for the somatic mutation theory of ageing (Cagan et al., 2022). By performing whole‐genome sequencing of colorectal crypt cells in 16 different species of mammals, Cagan et al. characterised the mutational landscape of these species. In particular, they reported a strong inverse relationship between somatic mutation rate per year and lifespan of the species. Their analysis highlighted that despite a 30‐fold variation in lifespan and a 40,000‐fold variation in body mass, the somatic mutation burden at the end of the lifespan varied only around a factor of 3, a finding that supports the somatic mutation theory of ageing (Cagan et al., 2022).

The strong negative correlation between lifespan and mean somatic mutation rate presented by Cagan et al. inspired us to explore seven additional phenotypic traits for predicting lifespan, that is, litter size, body mass, female and male sexual maturity, heart, respiratory, and mass‐specific basal metabolic rate (mass‐specific BMR). We hypothesised that (1) phylogenetic dependencies may impact lifespan predictions, (2) all phenotypic traits may explain a similar amount of lifespan variance, and (3) that a multifactorial model will ultimately enhance predictive power.

## 2 RESULTS

In this study, we obtained from Cagan et al. the lifespan, body weight, litter size, mass‐specific BMR, and somatic mutation rate of 15 mammalian species, that is, human, mouse, rat, giraffe, tiger, lion, cow, dog, cat, ferret, horse, black‐and‐white colobus, ring‐tailed lemur, and naked mole‐rat and rabbit. Harbour porpoise was excluded as this species lacked a sample age in Cagan et al. We complemented the phenotypic trait data with missing mass‐specific BMR, resting heart rate, respiratory rate estimates as well as female and male sexual maturity (Section 4; Table S1j). The selection of phenotypic traits used in our study occurred in a nonhypothesis driven manner, that is, determined by data availability. To mitigate the risk of biassed data collection, we leveraged phenotypic trait data from multiple studies when calculating the trait estimates (Section 4).

### 2.1 Phylogenetic dependencies of mammalian species

**FIGURE 1:**
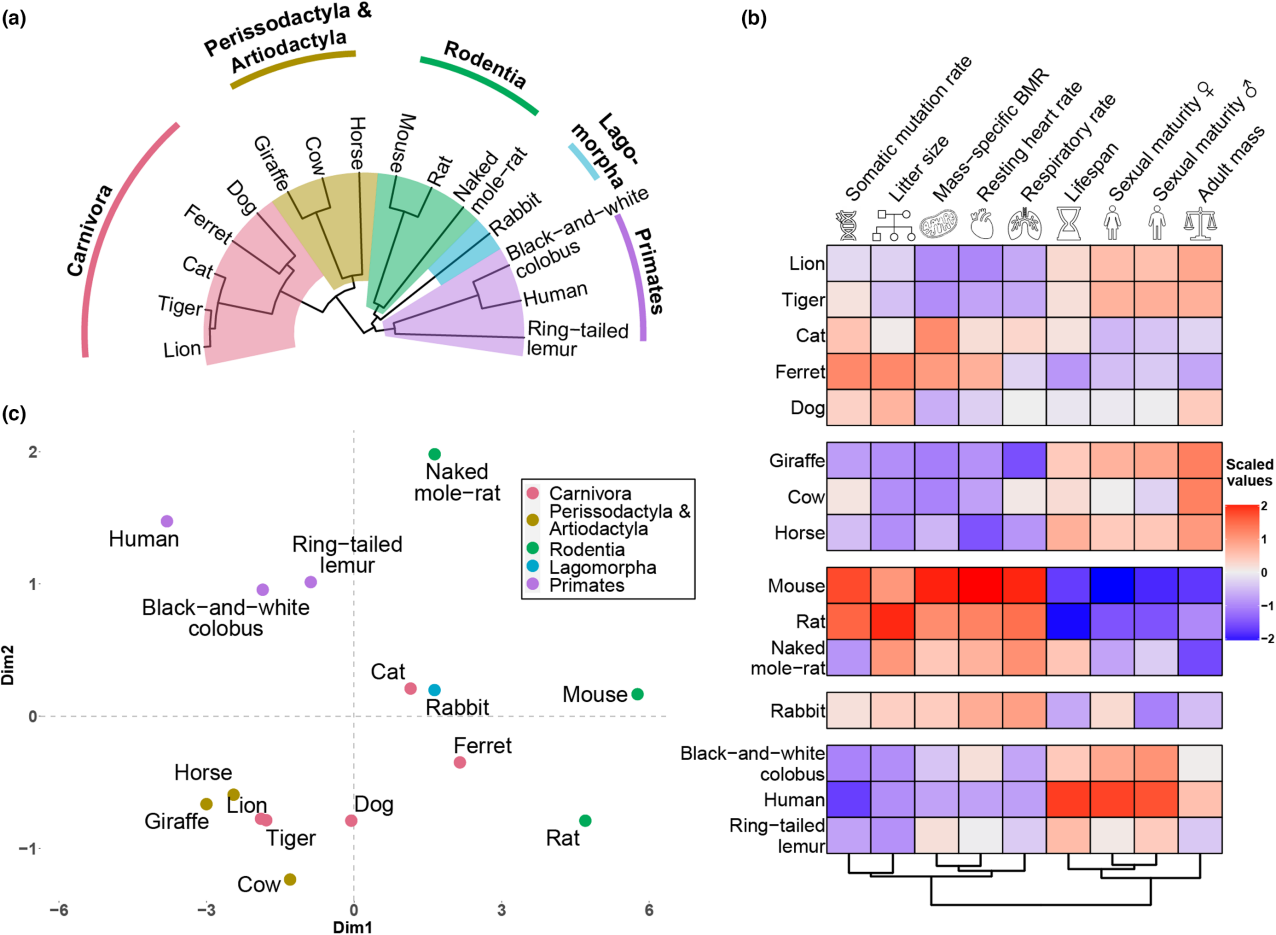
Characterisation of species. (a) Phylogenetic tree of the 15 species. The main branches corresponding to orders of species are indicated with different colours. (b) Heatmap of the data clustered by phenotypic traits (columns are scaled to Z‐scores), (c) Principal Component Analysis (PCA) plot of the data with all phenotypic traits included in the calculation of the principal components.

For highlighting the evolutionary relationship between the 15 mammalian species, we generated a phylogenetic tree based on DNA and fossil data (Figure 1a; Section 4) (Upham et al., 2019). The main branches corresponded to a set of mammalian orders, that is, *Perissodactyla and Artiodactyla*, *Carnivora*, *Rodentia*, *Lagomorpha*, *and Primates* (Vaughan et al., 2013). The differences between the branches were reflected in a clustered heatmap of the phenotypic traits (Figure 1b), where two main clusters of phenotypic traits were forming, which are anticorrelated with each other.

The clusters seen in the principal component analysis (PCA; Figure 1c) aligned well with the distances between the branches of the phylogenetic tree, whilst capturing expected outliers. For example, the naked mole‐rat is an outlier amongst *Rodentia*, which has been studied extensively due to its extraordinary lifespan (5‐times longer than expected by body mass) and apparent resilience to physiological decline that occurs in most mammals with ageing (Ruby et al., 2018).

First, we investigated the impact of phenotypic signals to predict lifespan, that is, predictive power of phenotypic similarity between species who share common ancestry (Revell et al., 2008). For this, we inspected the residuals from ordinary least squares (OLS) models and observed lacking patterns of autocorrelation (i.e., similar residuals of closely related species) and heteroscedasticity, which indicated no phylogenetic signal (Figure S1a–h) (Garamszegi, 2014; Revell, 2010). Furthermore, we found that fitting a phylogenetic generalised least squares model (PGLS), which assumes a strong phylogenetic signal, did not impact the residual distribution compared to the OLS model (Figures S1, S2). In addition, phylogenetic trees with low numbers of species (*n* < 20) are expected to have low power to detect phylogenetic signal (Freckleton et al., 2002), thus OLS linear modelling without considering phenotypic signals is the appropriate method following Revell's best practice (Revell, 2010).

### 2.2 Predicting lifespan based on univariate phenotypic traits

**FIGURE 2:**
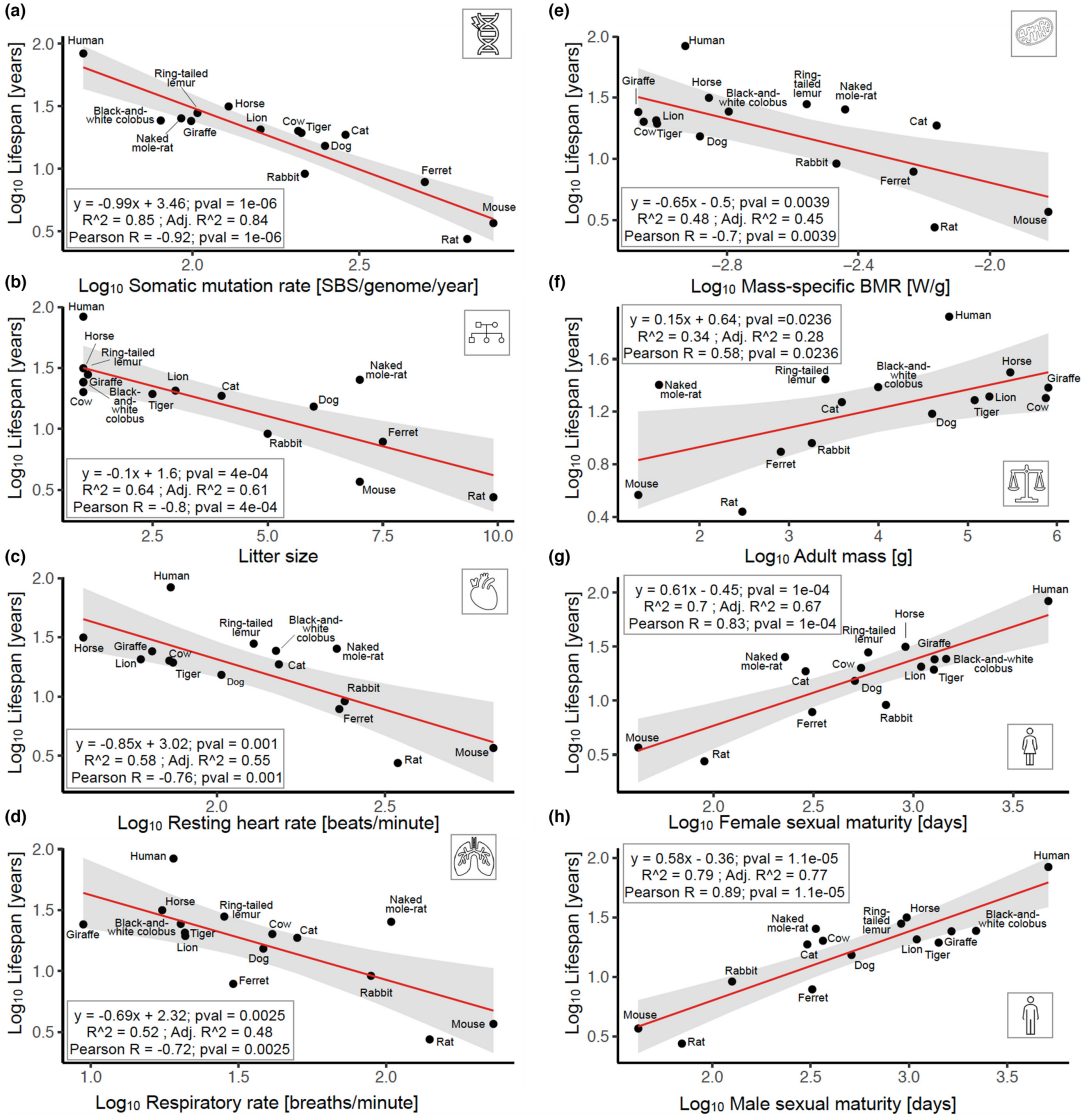
Phenotypic traits associated with lifespan. (a) Somatic mutation rate, (b) litter size, (c) resting heart rate, (d) respiratory rate, and (e) mass‐specific BMR are negatively correlated with lifespan. By contrast, (f) body mass, (g) female and (h) male sexual maturity are positively correlated with lifespan. All traits are significantly associated with lifespan, that is, five, two, and one with *p* < 0.001, *p* < 0.01, and *p* < 0.05, respectively.

First, we modelled lifespan as a function of individual phenotypic traits, which were extracted from multiple sources based on extensive literature search (Section 4). We observed significant correlations for each phenotypic trait (FDR <5%; Figure 2a–f; Table S4a), which is concordant with previous studies (de Magalhães et al., 2007; Levine, 1997; Ricklefs, 2010; Roderick & Storer, 1961; Speakman, 2005). Somatic mutation rate showed the strongest negative Pearson correlation, followed by litter size, resting heart rate, and respiratory rate (Figure 2a–e), whereas mass‐specific BMR, body mass, female and male time to sexual maturity displayed a positive correlation (Figure 2f–h). Although somatic mutation rate proved to be the most correlated trait (Figure 2a), we demonstrated that all other traits displayed significant cocorrelations with lifespan.

### 2.3 Multifactorial model to predict lifespan

**FIGURE 3:**
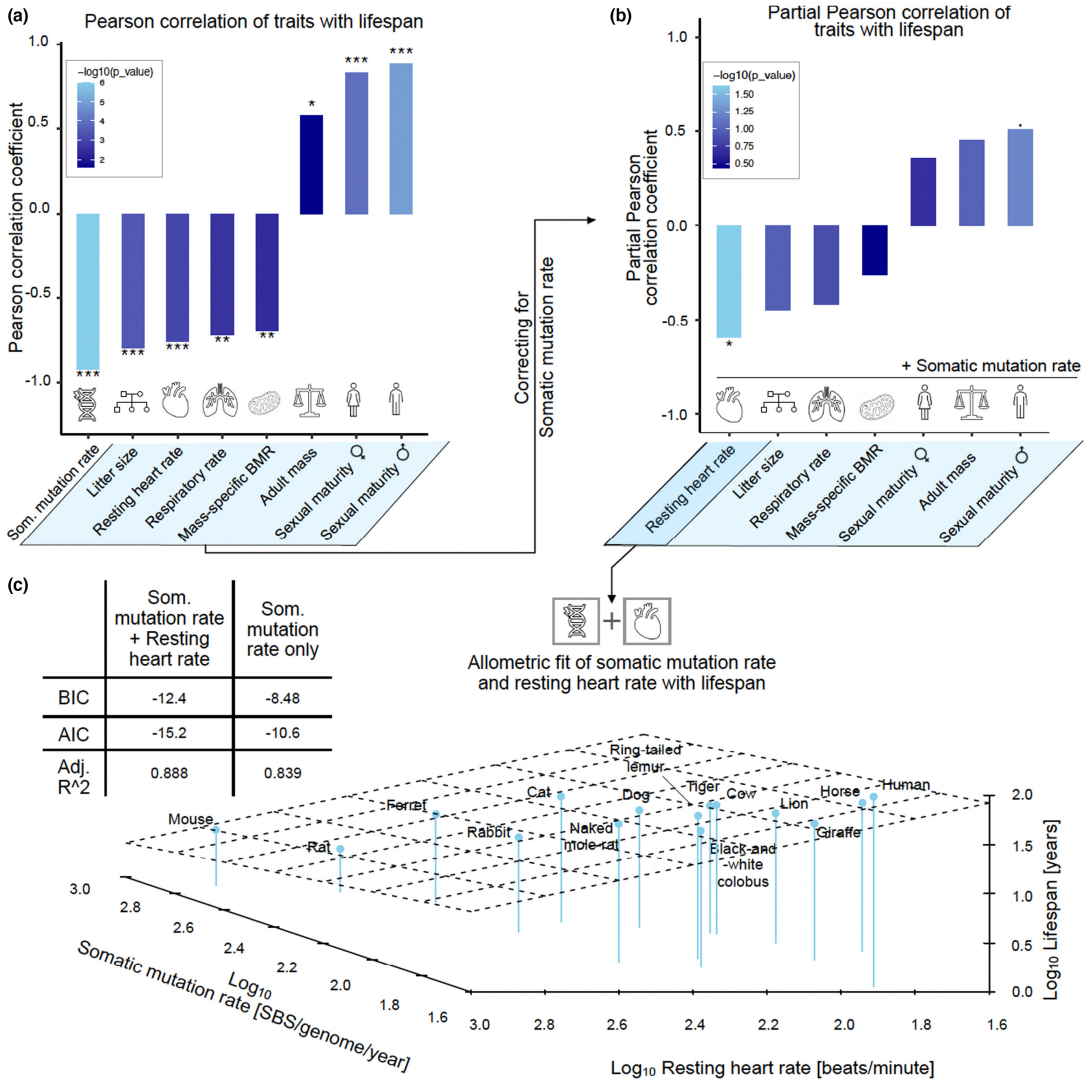
Combining heart and somatic mutation rate improves lifespan prediction. (a) Correlations of phenotypic traits with lifespan. (b) Partial correlation which corrects for somatic mutation rate. The corresponding significance levels of the correlation coefficients are visualised with, *, **, and *** if they are below 0.1, 0.05, 0.01, and 0.001, respectively. (c) Combining somatic mutation and resting heart rate in a multivariate regression model improves lifespan prediction from adj. *R* ^2^ = 0.84–adj. *R* ^2^ = 0.89.

In the light of the strong association of somatic mutation rate and lifespan (Figure 3a; Table S4a), we investigated if any other phenotypic trait improves predictions of lifespan. In order to address this, we performed partial correlation analysis where we removed the variation in lifespan explained by somatic mutation rate, and tested if any of the remaining traits can explain additional residual variance in lifespan (Figure 3b; Table S4b). Our results showed that resting heart rate was the only trait that explained significant further variance on top of somatic mutation rate (*p* = 0.0242; FDR < 20%; Table S4b).

To further investigate the benefits of including additional phenotypic traits, we compared a univariate linear regression model predicting lifespan from somatic mutation rate to multivariate regression models that include an additional trait as a second independent variable next to mean somatic mutation rate (Figure 3c; Table S4c). Leveraging OLS regression models we confirmed that resting heart rate was the only trait significantly improving the prediction of lifespan from adj. *R^2^* 0.84–0.89 (*p*‐value <0.05). In addition, we validated the stability of this finding based on bootstrapping (Section 4; Figure S16), underpinning that resting heart rate can explain additional variance in lifespan on top of somatic mutation rate.

## 3 DISCUSSION

In this study, we discovered that somatic mutation rate is the strongest predictor of mammalian lifespan. We investigated eight phenotypic traits across 15 mammalian species, and found that each trait was independently associated with lifespan, which is anticipated and concordant with literature (de Magalhães et al., 2007; Levine, 1997; Ricklefs, 2010; Roderick & Storer, 1961; Speakman, 2005). Reproducing established associations highlighted the reliability of our approach. In essence, we benchmarked the association strength of each phenotypic trait with lifespan, which revealed that the somatic mutation rate had the highest correlation.

The hallmark study of Cagan et al. (Cagan et al., 2022) provided us with the somatic mutation rate (Table S1), and observed an association between somatic mutation rate and lifespan (Cagan et al., 2022). However, Cagan et al. predicted the somatic mutation rate of mammalian species, whilst we predicted their lifespans. The distinct methodological difference is that we modelled lifespan as a dependent variable of phenotypic traits. In more detail, we predicted lifespan based on somatic mutation rate and/or other phenotypic traits. This enabled us to compare and jointly analyse phenotypic traits in the context of ageing, thus being complementary to the Cagan et al study.

Furthermore, we showed that the combination of somatic mutation rate and resting heart rate (RHR) best explained lifespan across mammals. By performing partial correlation analysis, we removed somatic mutation rate's effect from lifespan prediction and found that RHR was the only trait that explained additional variance. Therefore, integrating RHR and somatic mutation rate boosted the prediction of lifespan (adj. *R* ^2^ = 0.89) compared to a model containing somatic mutation rate alone (adj. *R* ^2^ = 0.84). These results demonstrated that resting heart rate may either directly influence lifespan, or be an epiphenomenon for one or more lower‐level mechanisms, which are complementary to somatic mutation rate.

Previous research found an inverse relationship between RHR and lifespan. Levine (Levine, 1997) demonstrated this inverse relationship in mammals and showed that the total number of heartbeats in a lifetime is within an order of magnitude across mammals despite the 40‐fold difference in their lifespan. Based on these results, a study conducted on mice (Gent et al., 2015) hypothesised that a decrease in resting heart rate should result in an equal growth in lifespan. The results showed a median lifespan increase of 6.2% by the administration of life‐long ivabradine (heart rate reduction 14%), underpinning the inverse relationship between RHR and lifespan, although the effect size was not translated in a 1:1 ratio. Similar values were observed in the prospective Copenhagen City Heart Study, where ~6500 healthy participants were followed for 18 years. Here, a 19% lower RHR was associated with an increase of life expectancy of 4.6 and 3.6 years for men and women, respectively (Jensen, 2019).

Research investigating heart rate's direct effects on mortality implicates that elevated heart rate plays a causal role in the development of atherosclerotic lesions and arterial stiffness via altered hemodynamics (Custodis et al., 2010). In humans, atherosclerosis has a strong impact on mortality, it represents the commonest cause of death (Varki et al., 2009), frequently affecting coronary arteries. However, this is not the case for other mammals, where signs of spontaneous atherosclerosis are present (Dangerfield et al., 1976; Finlayson et al., 1962), but it rarely appears in coronary arteries or leads to fatal events (Kawanishi et al., 2019). Rodents further corroborated this finding, as they show a relative resistance to atherosclerosis (Getz & Reardon, 2012; Ritskes‐Hoitinga & Beynen, 1988) despite having an elevated RHR. This highlights that although the inverse relationship between heart rate and lifespan seems to be universal among mammals, the exact mechanisms of how heart rate directly influences lifespan may vary between mammalian species.

Previous studies established that metabolic rate is a strong determinant of heart rate (Hulbert et al., 2007; Smith et al., 1976; Wolff, 2008). It has been shown that both at rest and during exercise cardiac output (Wolff, 2008) and therefore heart rate (Smith et al., 1976) are determined by metabolic rate (Smith et al., 1976), which is established to display a negative correlation with lifespan (Hulbert et al., 2007). More evidence of heart rate being a proxy for metabolic rate has been recently provided by Escala (Escala, 2019), who proposed that metabolic rate is a linear function of heart rate, body mass, and controlling factors (temperature and O_2_‐absportion). By leveraging this new metabolic rate relation and assuming that the total number of respiration cycles per lifetime across species is fixed (to generalise for animals without hearts), he showed empirical support for the rate of living theory (Escala, 2022), suggesting that basic energetics of respiration predetermine lifespan via a fixed number of cardiorespiratory cycles per lifetime. These findings strongly support the fact that heart rate can be viewed as a proxy for metabolic rate, and being valid for resting conditions, that is, RHR ∝ basal metabolic rate. This may drive the correlation between resting heart rate and lifespan after correcting for somatic mutation rate's effect.

Resting heart rate (RHR) was the only significantly correlated trait with lifespan after correcting for somatic mutation rate. It might be surprising that mass‐specific basal metabolic rate (BMR) did not achieve significance after correction (Figure 3b), since there is a strong causal linear relationship between mass‐specific BMR and RHR (Escala, 2019; Smith et al., 1976; Wolff, 2008). We also validated the stability of this finding based on bootstrapping (Section 4; Figure S17). Therefore, we reasoned that RHR must contain a metabolic rate‐driven and metabolic rate‐independent component contributing to lifespan prediction.

We hypothesise the metabolic rate‐driven component of RHR is regressed out by correcting for somatic mutation rate. Mass‐specific BMR is known to affect further cellular processes parallel to determining heart rate: it shows linear correlation with oxidative DNA damage in mammals (Adelman et al., 1988), possibly contributing to a specific somatic mutational signature (Alexandrov et al., 2020) via reactive oxygen species (ROS) (Martin & Palumbi, 1993), and is also known to affect lifespan via oxidative damage to membrane lipids and proteins (Hamon et al., 2015; Hulbert et al., 2007; Juan et al., 2021). By removing the effect of somatic mutation rate, the correlation of mass‐specific BMR with lifespan is diminished.

Metabolic rate‐independent correlates of RHR that display correlation with lifespan have been identified in human population studies, that is, blood pressure, physical activity, sleep, and smoking (Olshansky et al., 2022; Rabbia et al., 2002; Zhang & Kesteloot, 1999). As these correlations may not necessarily generalise to all mammals and lack deep mechanistic understanding, further research is needed to verify and discover novel metabolic rate‐independent phenomena that contribute both to RHR and lifespan.

Limitations of our study came from data sparsity: as only recent technological developments made it possible to assess somatic mutations in healthy tissues (Cagan et al., 2022), thus the number of species with available somatic mutation rate data in the public domain is limited (Cagan et al., 2022). As sequencing technology is getting more affordable, larger datasets in the near future will raise statistical power and provide the opportunity to generalise our analysis to other animal classes. Another aspect of data sparsity was the unbalanced phenotypic characterisation between species, which limited the number of accessible phenotypic traits. We mitigated the risk of biassed data collection by leveraging phenotypic trait data from multiple studies and calculating ensemble estimates, where the unbalanced number of subjects between species and studies may constitute a limiting factor for phenotypic trait calculations.

Another challenge we encountered was the lack of standardisation. Heart rate variability (HRV) is reflecting the long‐term (i.e., circadian cycle, core body temperature, and metabolism) and short‐term (i.e., autonomic, cardiovascular, and respiratory system) regulatory mechanisms that control heart rate (Shaffer & Venner, 2013) and has been linked to human longevity (Hernández‐Vicente et al., 2020) and postinfarction mortality (Buccelletti et al., 2009). Human HRV measurements are well standardised enforcing compatibility (Malik, 1996) and are available in abundance. However, data and standardisation was lacking for most other mammalian species included in our study (García‐González et al., 2009; Manzo et al., 2009), thus HRV was not considered. It is our belief that RHR captures all information content of HRV, since HRV provides information about controlling mechanisms of heart rate. This is supported by strong linear correlations observed between HRV and heart rate in humans (Kazmi et al., 2016) and rats (Kazmi et al., 2016; Mangin et al., 1998).

Further challenges are that correlations do not imply causation. The low number of species and sparse trait data permitted the identification of significant correlations, however, causal inference and gaining mechanistic insights remains challenging. In order to detangle the relationships between high‐level traits used in this study and to obtain a deeper understanding of the processes of ageing, inclusion of further lower‐level mechanisms would be necessary.

In summary, we found that all phenotypic traits were significantly associated with lifespan, whilst somatic mutation rate was the most significant one. Interestingly, integrating somatic mutation and resting heart rate became the most predictive model of lifespan, which underpinned that ageing is multifactorial. We hypothesise that resting heart rate is partially driven by metabolic rate and a metabolic rate‐independent component. Nevertheless, for detangling correlations and causality, further mechanistic studies are required to derive a global theory of ageing, which ultimately may pave the way to prolong human lifespans.

## 4 METHODS

### 4.1 Data acquisition

Lifespan data was obtained from Cagan et al. (Cagan et al., 2022) Species 360/Human Mortality Database. Identical to the original publication, the age at which 80% of the species had died was used as a proxy for lifespan, excluding infant mortality. To remove the effect of extrinsic mortality, the data were acquired for individuals under human care. Values for litter size, adult mass, mass‐specific basal metabolic rate, female and male sexual maturity were mostly extracted from the established AnAge consensus database (de Magalhães & Costa, 2009) (Table S1) in concordance with Cagan et al. (Cagan et al., 2022). Exceptions lacking AnAge data are male sexual maturity of rabbits and naked mole rats, which were extracted from literature (Table S1). Mass‐specific basal metabolic rate of six species (ferret, dog, cat, giraffe, ring‐tailed lemur, and horse) were also lacking AnAge data, therefore were completed with literature estimates based on three to 11 individuals per species (Table [Link], [Link]). The Cagan et al. study provided somatic mutation rate, leveraging at least one individual per species (Cagan et al., 2022). Estimates of resting heart rate and respiratory rate were extracted from up to 12 independent sources per species resulting in a minimum of eight and up to 12,147 individuals for calculating these phenotypic traits (Section 4; Tables S1c,d, S2). Detailed summary statistics of all phenotypic traits, their respective sources and number of individuals are contained in Tables S1–S3.

Most studies used for the calculation of estimates were not reporting the exact age of subjects. To mitigate the risk of age bias, we only used data of adult animals ranging from early to late adulthood, thereby increasing the reliability of estimates. To increase comparability between studies, phenotypic traits were estimated based on control group animals in resting states. For species posing danger to humans, for example, *tiger or lion*, the resting state data were derived from sedated subjects.

### 4.2 Heart and respiratory rate

To estimate heart and respiratory rate, we collected mean heart and respiratory rates from multiple publications (Table S2). Between eight and > 12 k individuals per species were leveraged to estimate heart and respiratory rate (Table S2). The exception is the black‐and‐white colobus where due to a lack of data we used estimates based on its average weight (Table S1). We calculated the weighted mean and standard deviation over the collected heart and respiratory rates according to the number of subjects measured per study group.

### 4.3 Mass‐specific basal metabolic rate (BMR)

The majority of metabolic data was collected from the AnAge database (de Magalhães & Costa, 2009) as mass‐specific basal metabolic rate (*W/g*). Estimates for mass‐specific BMR of species not available in the AnAge database (Table S3) were collected from other studies, that is, ring‐tailed lemur, calculated by converting mass‐specific basal oxygen consumption to energy units via the conversion rate *1 Litre O*_*2*_ *= 20.1 kJ (*Escala, 2022 *)* (i.e., *dog*, *horse*, *and giraffe)* or converted mass‐specific metabolic rates from W/kg^0.75^ and kcal/kg^0.75^/day units to W/g using the weight of the studied animals (i.e., *ferret*) or the average weight of the studied group of animals (i.e., *cat*). At least three individuals were assessed per species (Table S3). In order to address variation in the mass of the different dog species we took the metabolic rate estimates of the grey wolf (*Canis lupus*), hence according to AnAge database the domesticated dog is the descendant of the grey wolf and is “technically not an individual species” (de Magalhães & Costa, 2009). The average mass of the grey wolf group we used for estimation (~35 kg) is close to the adult mass of the domestic dog (~40 kg) taken from AnAge (de Magalhães & Costa, 2009).

### 4.4 Phylogenetic tree construction

We acquired credible sets of phylogenetic trees (10,000 trees containing the 15 species assessed in this paper) as a single nexus file from vertlife.org, which is a platform for downloading subsets of mammalian phylogenetic trees created by Upham et al. (2019) using DNA and fossil data. We chose “*Birth‐death tip‐dated complemented trees”* from vertlife.org, as this leverages both DNA and fossil data for creating the tips of the phylogenetic trees (Upham et al., 2019). For the construction of a consensus phylogenetic tree from the 10,000 trees we used the *consensus. edge()* function of the *phytools R* package. For further analysis, we rooted the consensus tree by the midpoint rooting method, as the tree is balanced and lacking established outgroups (Kinene et al., 2016).

### 4.5 Phylogenetically corrected linear model

For our analysis we used *caper* R package. We compiled the rooted tree and the phenotypic dataset into a comparative dataset for *caper* and fitted a phylogenetic generalised least squares model with the *pgls()* with strong phylogenetic signal.

### 4.6 Statistical analysis

Statistical analyses were conducted in *R*. For the log_10_–log_10_ allometric regression models, a linear model *lm()* was fitted to the log_10_ values of the specific trait and the specie's lifespan, respectively. Pearson correlations and partial correlations were obtained from log_10_ values, with the exception of litter size. The computations were executed with *cor.test()* and *pcor.test()* using the Pearson method. All *p*‐values were obtained from two‐tailed *t* tests. We corrected for multiple testing using the Benjamini–Hochberg method. The final predictive model for the log_10_ lifespan using the mutation rate and heart beat in the log_10_ space was fitted with *lm()* as well and evaluated with the *broom* library.

### 4.7 Bootstrapping analysis

We created a set of bootstrapped data by resampling our original dataset *n =* 1000 times with replacement. For this we use the *sample_n()* function of the *dplyr* package in *R*, setting the *“size”* parameter to the number of species used in the study (*n* = 15). Further analyses performed on the bootstrapped datasets were carried out in the same manner as described in the Section 4.6.

## AUTHOR CONTRIBUTIONS

D.G., M.M. and M.P.M. were involved in conceptualization; D.G. and T.D. were involved in data curation; D.G., M.M. and M.P.M were involved in analysis; D.G., M.M. and M.P.M were involved in methodology; M.P.M. was involved in supervision; D.G., M.M. and T.D. were involved in visualisation; D.G., M.M. and M.P.M. were involved in writing original draft; all authors were involved in writing original draft.

## FUNDING INFORMATION

This project was supported by the European Union's Horizon 2020 Research and Innovation Programme (Grant agreement No. 950293‐COMBAT‐RES). M.M. is supported by the Helmholtz Association under the joint research school "Munich School for Data Science ‐ MUDS".

## CONFLICT OF INTEREST STATEMENT

M.P.M. collaborates with GSK, Roche and AstraZeneca, and receives funding from Roche and GSK. M.P.M. is a former employee at AstraZeneca.

## CODE ACCESSIBILITY

The source code to reproduce all results, supplements, and figures are accessible under the following link: https://github.com/MendenLab/cardiovascularlifespan

## Supporting information


Appendix S1

Table S1

Table S2

Table S3

Table S4

## Data Availability

The data of Cagan et al. (2022) is deposited in Zenodo https://doi.org/10.5281/zenodo.5554777. Phenotypic characterisation and sources are stored in Tables [Link], [Link], [Link].

## References

[acel13957-bib-0001] Adelman, R. , Saul, R. L. , & Ames, B. N. (1988). Oxidative damage to DNA: Relation to species metabolic rate and life span. Proceedings of the National Academy of Sciences of the United States of America, 85, 2706–2708. 3128794 10.1073/pnas.85.8.2706 PMC280067

[acel13957-bib-0002] Alexandrov, L. B. , Kim, J. , Haradhvala, N. J. , Huang, M. N. , Tian Ng, A. W. , Wu, Y. , Boot, A. , Covington, K. R. , Gordenin, D. A. , Bergstrom, E. N. , Islam, S. M. A. , Lopez‐Bigas, N. , Klimczak, L. J. , McPherson, J. R. , Morganella, S. , Sabarinathan, R. , Wheeler, D. A. , Mustonen, V. , PCAWG Mutational Signatures Working Group , … PCAWG Consortium . (2020). The repertoire of mutational signatures in human cancer. Nature, 578, 94–101. 32025018 10.1038/s41586-020-1943-3 PMC7054213

[acel13957-bib-0003] Balcombe, N. R. , & Sinclair, A. (2001). Ageing: Definitions, mechanisms and the magnitude of the problem. Best Practice & Research. Clinical Gastroenterology, 15, 835–849. 11866480 10.1053/bega.2001.0244

[acel13957-bib-0004] Buccelletti, E. , Gilardi, E. , Scaini, E. , Galiuto, L. , Persiani, R. , Biondi, A. , Basile, F. , & Silveri, N. G. (2009). Heart rate variability and myocardial infarction: Systematic literature review and metanalysis. European Review for Medical and Pharmacological Sciences, 13, 299–307. 19694345

[acel13957-bib-0005] Cagan, A. , Baez‐Ortega, A. , Brzozowska, N. , Abascal, F. , Coorens, T. H. H. , Sanders, M. A. , Lawson, A. R. J. , Harvey, L. M. R. , Bhosle, S. , Jones, D. , Alcantara, R. E. , Butler, T. M. , Hooks, Y. , Roberts, K. , Anderson, E. , Lunn, S. , Flach, E. , Spiro, S. , Januszczak, I. , … Martincorena, I. (2022). Somatic mutation rates scale with lifespan across mammals. Nature, 604, 517–524. 35418684 10.1038/s41586-022-04618-z PMC9021023

[acel13957-bib-0006] Custodis, F. , Schirmer, S. H. , Baumhäkel, M. , Heusch, G. , Böhm, M. , & Laufs, U. (2010). Vascular pathophysiology in response to increased heart rate. Journal of the American College of Cardiology, 56, 1973–1983. 21126638 10.1016/j.jacc.2010.09.014

[acel13957-bib-0007] Dangerfield, W. G. , Finlayson, R. , Myatt, G. , & Mead, M. G. (1976). Serum lipoproteins and atherosclerosis in animals. Atherosclerosis, 25, 95–106. 186081 10.1016/0021-9150(76)90051-4

[acel13957-bib-0008] de Magalhães, J. P. , & Costa, J. (2009). A database of vertebrate longevity records and their relation to other life‐history traits. Journal of Evolutionary Biology, 22, 1770–1774. 19522730 10.1111/j.1420-9101.2009.01783.x

[acel13957-bib-0009] de Magalhães, J. P. , Costa, J. , & Church, G. M. (2007). An analysis of the relationship between metabolism, developmental schedules, and longevity using phylogenetic independent contrasts. The Journals of Gerontology. Series A, Biological Sciences and Medical Sciences, 62, 149–160. 17339640 10.1093/gerona/62.2.149 PMC2288695

[acel13957-bib-0010] Escala, A. (2019). The principle of similitude in biology. Theoretical Ecology, 12, 415–425.

[acel13957-bib-0011] Escala, A. (2022). Universal relation for life‐span energy consumption in living organisms: Insights for the origin of aging. Scientific Reports, 12, 2407. 35190571 10.1038/s41598-022-06390-6 PMC8861023

[acel13957-bib-0012] Finlayson, R. , Symons, C. , & Fiennes, R. N. (1962). Atherosclerosis: A comparative study. British Medical Journal, 1, 501–507. 13893013 10.1136/bmj.1.5277.501 PMC1958245

[acel13957-bib-0013] Freckleton, R. P. , Harvey, P. H. , & Pagel, M. (2002). Phylogenetic analysis and comparative data: A test and review of evidence. The American Naturalist, 160, 712–726. 10.1086/343873 18707460

[acel13957-bib-0014] Garamszegi, L. Z. (2014). Modern Phylogenetic Comparative Methods And Their Application In Evolutionary Biology. Springer Berlin Heidelberg.

[acel13957-bib-0015] García‐González, M. A. , Fernández‐Chimeno, M. , Escorihuela, R. , & Ramos‐Castro, J. (2009). An empirical methodology for the definition of frequency bands for spectral analysis of heart rate. In World Congress On Medical Physics And Biomedical Engineering (Vol. 25, pp. 449–452). Springer Publishing.

[acel13957-bib-0016] Gent, S. , Kleinbongard, P. , Dammann, P. , Neuhäuser, M. , & Heusch, G. (2015). Heart rate reduction and longevity in mice. Basic Research in Cardiology, 110, 2. 25589054 10.1007/s00395-014-0460-7

[acel13957-bib-0017] Getz, G. S. , & Reardon, C. A. (2012). Animal models of atherosclerosis. Arteriosclerosis, Thrombosis, and Vascular Biology, 32, 1104–1115. 22383700 10.1161/ATVBAHA.111.237693 PMC3331926

[acel13957-bib-0018] Hamon, M.‐P. , Bulteau, A.‐L. , & Friguet, B. (2015). Mitochondrial proteases and protein quality control in ageing and longevity. Ageing Research Reviews, 23, 56–66. 25578288 10.1016/j.arr.2014.12.010

[acel13957-bib-0019] Harley, C. B. (1991). Telomere loss: Mitotic clock or genetic time bomb? Mutation Research, 256, 271–282. 1722017 10.1016/0921-8734(91)90018-7

[acel13957-bib-0020] Harman, D. (1956). Aging: A theory based on free radical and radiation chemistry. Journal of Gerontology, 11, 298–300. 13332224 10.1093/geronj/11.3.298

[acel13957-bib-0021] Harman, D. (2003). The free radical theory of aging. Antioxidants & Redox Signaling, 5, 557–561. 14580310 10.1089/152308603770310202

[acel13957-bib-0022] Hernández‐Vicente, A. , Hernando, D. , Santos‐Lozano, A. , Rodríguez‐Romo, G. , Vicente‐Rodríguez, G. , Pueyo, E. , Bailón, R. , & Garatachea, N. (2020). Heart rate variability and exceptional longevity. Frontiers in Physiology, 11, 566399. 33041862 10.3389/fphys.2020.566399 PMC7527628

[acel13957-bib-0023] Hulbert, A. J. , Pamplona, R. , Buffenstein, R. , & Buttemer, W. A. (2007). Life and death: Metabolic rate, membrane composition, and life span of animals. Physiological Reviews, 87, 1175–1213. 17928583 10.1152/physrev.00047.2006

[acel13957-bib-0024] Jensen, M. T. (2019). Resting heart rate and relation to disease and longevity: Past, present and future. Scandinavian Journal of Clinical and Laboratory Investigation, 79, 108–116. 30761923 10.1080/00365513.2019.1566567

[acel13957-bib-0025] Juan, C. A. , Pérez de la Lastra, J. M. , Plou, F. J. , & Pérez‐Lebeña, E. (2021). The chemistry of reactive oxygen species (ROS) revisited: Outlining their role in biological macromolecules (DNA, lipids and proteins) and induced pathologies. International Journal of Molecular Sciences, 22, 4642. 33924958 10.3390/ijms22094642 PMC8125527

[acel13957-bib-0026] Kanungo, M. S. (1975). A model for ageing. Journal of Theoretical Biology, 53, 253–261. 1195761 10.1016/s0022-5193(75)80002-6

[acel13957-bib-0027] Kawanishi, K. , Dhar, C. , Do, R. , Varki, N. , Gordts, P. L. S. M. , & Varki, A. (2019). Human species‐specific loss of CMP‐*N*‐acetylneuraminic acid hydroxylase enhances atherosclerosis via intrinsic and extrinsic mechanisms. Proceedings of the National Academy of Sciences of the United States of America, 116, 16036–16045. 31332008 10.1073/pnas.1902902116 PMC6690033

[acel13957-bib-0028] Kazmi, S. Z. H. , Zhang, H. , Aziz, W. , Monfredi, O. , Abbas, S. A. , Shah, S. A. , Kazmi, S. S. H. , & Butt, W. H. (2016). Inverse correlation between heart rate variability and heart rate demonstrated by linear and nonlinear analysis. PLoS One, 11, e0157557. 27336907 10.1371/journal.pone.0157557 PMC4919077

[acel13957-bib-0029] Kinene, T. , Wainaina, J. , Maina, S. , & Boykin, L. M. (2016). Rooting Trees, Methods for. Encyclopedia of Evolutionary Biology, 3, 489.

[acel13957-bib-0030] Kirkwood, T. B. (1977). Evolution of ageing. Nature, 270, 301–304. 593350 10.1038/270301a0

[acel13957-bib-0031] Kowald, A. , & Kirkwood, T. B. (1996). A network theory of ageing: The interactions of defective mitochondria, aberrant proteins, free radicals and scavengers in the ageing process. Mutation Research, 316, 209–236. 8649456 10.1016/s0921-8734(96)90005-3

[acel13957-bib-0032] Levine, H. J. (1997). Rest heart rate and life expectancy. Journal of the American College of Cardiology, 30, 1104–1106. 9316546 10.1016/s0735-1097(97)00246-5

[acel13957-bib-0033] López‐Otín, C. , Blasco, M. A. , Partridge, L. , Serrano, M. , & Kroemer, G. (2013). The hallmarks of aging. Cell, 153, 1194–1217. 23746838 10.1016/j.cell.2013.05.039 PMC3836174

[acel13957-bib-0034] Malik, M. (1996). Heart rate variability. Annals of Noninvasive Electrocardiology, 1, 151–181.

[acel13957-bib-0035] Mangin, L. , Swynghedauw, B. , Benis, A. , Thibault, N. , Lerebours, G. , & Carré, F. (1998). Relationships between heart rate and heart rate variability: Study in conscious rats. Journal of Cardiovascular Pharmacology, 32, 601–607. 9781928 10.1097/00005344-199810000-00012

[acel13957-bib-0036] Manzo, A. , Ootaki, Y. , Ootaki, C. , Kamohara, K. , & Fukamachi, K. (2009). Comparative study of heart rate variability between healthy human subjects and healthy dogs, rabbits and calves. Laboratory Animals, 43, 41–45. 19001066 10.1258/la.2007.007085

[acel13957-bib-0037] Marchionni, S. , Sell, C. , & Lorenzini, A. (2020). Development and longevity: Cellular and molecular determinants—A mini‐review. Gerontology, 66, 223–230. 32036369 10.1159/000505327

[acel13957-bib-0038] Martin, A. P. , & Palumbi, S. R. (1993). Body size, metabolic rate, generation time, and the molecular clock. Proceedings of the National Academy of Sciences of the United States of America, 90, 4087–4091. 8483925 10.1073/pnas.90.9.4087 PMC46451

[acel13957-bib-0039] Morley, A. A. (1995). The somatic mutation theory of ageing. Mutation Research, 338, 19–23. 7565875 10.1016/0921-8734(95)00007-s

[acel13957-bib-0040] Olshansky, B. , Ricci, F. , & Fedorowski, A. (2022). Importance of resting heart rate. Trends in Cardiovascular Medicine, S1050‐1738, 00073. 10.1016/j.tcm.2022.05.006 35623552

[acel13957-bib-0041] Orgel, L. E. (1963). The maintenance of the accuracy of protein synthesis and its relevance to ageing. Proceedings of the National Academy of Sciences of the United States of America, 49, 517–521. 13940312 10.1073/pnas.49.4.517 PMC299893

[acel13957-bib-0042] Pearl, R. (1928). The Rate of Living. University of London Press.

[acel13957-bib-0043] Proshkina, E. N. , Solovev, I. A. , Shaposhnikov, M. V. , & Moskalev, A. A. (2020). Key molecular mechanisms of aging, biomarkers, and potential interventions. Molecular Biology, 54, 777–811. 10.31857/S0026898420060099 33276355

[acel13957-bib-0044] Rabbia, F. , Grosso, T. , Cat Genova, G. , Conterno, A. , De Vito, B. , Mulatero, P. , Chiandussi, L. , & Veglio, F. (2002). Assessing resting heart rate in adolescents: Determinants and correlates. Journal of Human Hypertension, 16, 327–332. 12082493 10.1038/sj.jhh.1001398

[acel13957-bib-0045] Revell, L. J. (2010). Phylogenetic signal and linear regression on species data. Methods in Ecology and Evolution, 1, 319–329.

[acel13957-bib-0046] Revell, L. J. , Harmon, L. J. , & Collar, D. C. (2008). Phylogenetic signal, evolutionary process, and rate. Systematic Biology, 57, 591–601. 18709597 10.1080/10635150802302427

[acel13957-bib-0047] Ricklefs, R. E. (2010). Life‐history connections to rates of aging in terrestrial vertebrates. Proceedings of the National Academy of Sciences of the United States of America, 107, 10314–10319. 20479246 10.1073/pnas.1005862107 PMC2890449

[acel13957-bib-0048] Ritskes‐Hoitinga, J. , & Beynen, A. C. (1988). Atherosclerosis in the rat. Artery, 16, 25–50. 3061372

[acel13957-bib-0049] Robin Harris, J. , & Korolchuk, V. I. (2019). Biochemistry and Cell Biology of Ageing: Part Ii Clinical Science. Springer Nature Singapore.

[acel13957-bib-0050] Roderick, T. H. , & Storer, J. B. (1961). Correlation between mean litter size and mean life span among 12 inbred strains of mice. Science, 134, 48–49. 13742527 10.1126/science.134.3471.48

[acel13957-bib-0051] Rubner, M. (1908). Das Problem der Lebensdauer und seiner beziehungen zum Wachstum und Ernährung. Ouldenburg.

[acel13957-bib-0052] Ruby, J. G. , Smith, M. , & Buffenstein, R. (2018). Naked mole‐rat mortality rates defy gompertzian laws by not increasing with age. eLife, 7, e31157. 29364116 10.7554/eLife.31157 PMC5783610

[acel13957-bib-0053] Shaffer, F. , & Venner, J. (2013). Heart rate variability anatomy and physiology. Biofeedback and Self‐Regulation, 41, 13–25.

[acel13957-bib-0054] Smith, E. E. , Guyton, A. C. , Manning, R. D. , & White, R. J. (1976). Integrated mechanisms of cardiovascular response and control during exercise in the normal human. Progress in Cardiovascular Diseases, 18, 421–444. 778915 10.1016/0033-0620(76)90010-4

[acel13957-bib-0055] Speakman, J. R. (2005). Body size, energy metabolism and lifespan. The Journal of Experimental Biology, 208, 1717–1730. 15855403 10.1242/jeb.01556

[acel13957-bib-0056] Timiras, P. S. (2007). Physiological Basis Of Aging And Geriatrics. CRC Press.

[acel13957-bib-0057] Upham, N. S. , Esselstyn, J. A. , & Jetz, W. (2019). Inferring the mammal tree: Species‐level sets of phylogenies for questions in ecology, evolution, and conservation. PLoS Biology, 17, e3000494. 31800571 10.1371/journal.pbio.3000494 PMC6892540

[acel13957-bib-0058] Varki, N. , Anderson, D. , Herndon, J. G. , Pham, T. , Gregg, C. J. , Cheriyan, M. , Murphy, J. , Strobert, E. , Fritz, J. , Else, J. G. , & Varki, A. (2009). Heart disease is common in humans and chimpanzees, but is caused by different pathological processes. Evolutionary Applications, 2, 101–112. 25567850 10.1111/j.1752-4571.2008.00064.x PMC3352420

[acel13957-bib-0059] Vaughan, T. A. , Ryan, J. M. , & Czaplewski, N. J. (2013). Mammalogy. Jones & Bartlett Learning.

[acel13957-bib-0060] Vijg, J. (2000). Somatic mutations and aging: A re‐evaluation. Mutation Research, 447, 117–135. 10686308 10.1016/s0027-5107(99)00202-x

[acel13957-bib-0061] Weinert, B. T. , & Timiras, P. S. (2003). Invited review: Theories of aging. Journal of Applied Physiology, 95, 1706–1716. 12970376 10.1152/japplphysiol.00288.2003

[acel13957-bib-0062] Williams, G. C. (1957). Pleiotropy, natural selection, and the evolution of senescence. Evolution, 11, 398–411.

[acel13957-bib-0063] Wolff, C. B. (2008). Normal cardiac output, oxygen delivery and oxygen extraction. In Oxygen Transport to Tissue Xxviii (pp. 169–182). Springer US. 10.1007/978-0-387-71764-7_23 17727262

[acel13957-bib-0064] Zhang, J. , & Kesteloot, H. (1999). Anthropometric, lifestyle and metabolic determinants of resting heart rate. A population study. European Heart Journal, 20, 103–110. 10099906 10.1053/euhj.1999.1230

[acel13957-bib-0065] Zhao, Y. , Seluanov, A. , & Gorbunova, V. (2021). Revelations about aging and disease from unconventional vertebrate model organisms. Annual Review of Genetics, 55, 135–159. 10.1146/annurev-genet-071719-021009 PMC8903061 34416119

