# Supplementary material for: The impact of the cardiovascular component and somatic mutations on ageing
Source: Aging Cell. 2023 Aug 22;22(10):e13957. doi: 10.1111/acel.13957 (PMC10577550; doi:10.1111/acel.13957)
Supplement: Supplementary file 1 — Appendix S1 [file ACEL-22-e13957-s003.docx]

**The impact of the cardiovascular component and somatic mutation on ageing**Daniel Garger, Martin Meinel, Tamina Dietl, Christina Hillig, Natalie Garzorz-Stark, Kilian Eyerich, Martin Hrabě de Angelis, Stefanie Eyerich, Michael P. Menden.

**Description of supplementary tables:**

***Supplementary Table 1: Characterisation of phenotypic traits.*** *This table contains data and their literature sources for (****a****) lifespan, (****b)*** *somatic mutation rate, (****c****) resting heart rate, (****d****) respiratory rate, (****e****) adult mass, (****f****) female sexual maturity, (****g****) male sexual maturity, (****h****) litter size, (****i****) mass-specific basal metabolic rate. (****j****) Contains all the previous data compiled in one table.*

***Supplementary Table 2: Calculation of resting heart rate and respiratory rate estimates for each species.*** *This table contains literature sources and the calculation of resting heart rate and respiratory rate estimates for the (****a****) rat, (****b****) mouse, (****c****) ferret, (****d****) rabbit, (****e****) dog, (****f****) tiger, (****g****) cow, (****h****) lion, (****i****) giraffe, (****j****) naked mole-rat, (****k****) ring-tailed lemur, (****l****) horse. Calculation of respiratory rate of the human is shown in (****m****).*

***Supplementary Table 3: Calculation of mass-specific basal metabolic rate estimates****. The table contains literature sources and the calculation of mass-specific basal metabolic rate estimates for the (****a****) ferret, (****b****) dog, (****c****) cat, (****d****) giraffe, (****e****) ring-tailed lemur and (****f****) horse****.***

***Supplementary Table 4: Results of statistical analysis.*** *This table contains the results of (****a****) correlation analysis, (****b****) partial correlation analysis and (****c****) linear model fits.*

**Supplementary figures:**

***Supplementary figure 1: Log lifespan as a function of log somatic mutation rate.*** *Here is shown the (****a****) QQplot, (****b****) residual and (****c****) scale-location plot of the univariate ordinary least squares (OLS) model. This model leverages lifespan as a function of somatic mutation rate, assuming statistical independence between the species (Pagel's lambda=0). In addition, we display the (****d****) QQplot,* *(****e****) residual and (****f****) scale-location plot of the univariate phylogenetic generalised least squares model (PGLS) model. This model uses lifespan as a function of somatic mutation rate, assuming a Brownian-motion model of evolution and a strong phylogenetic signal (Pagel's lambda=1).*
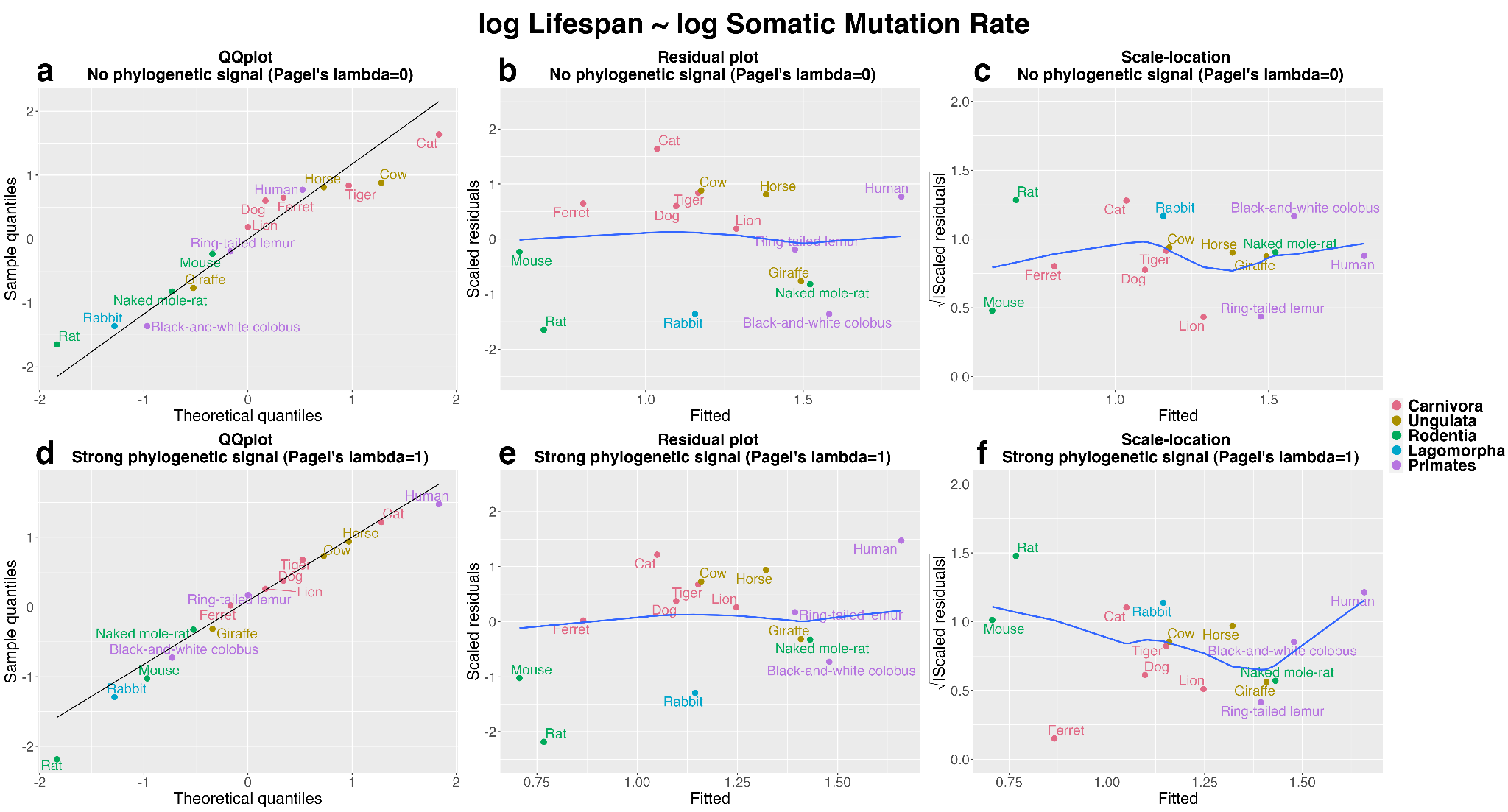


*
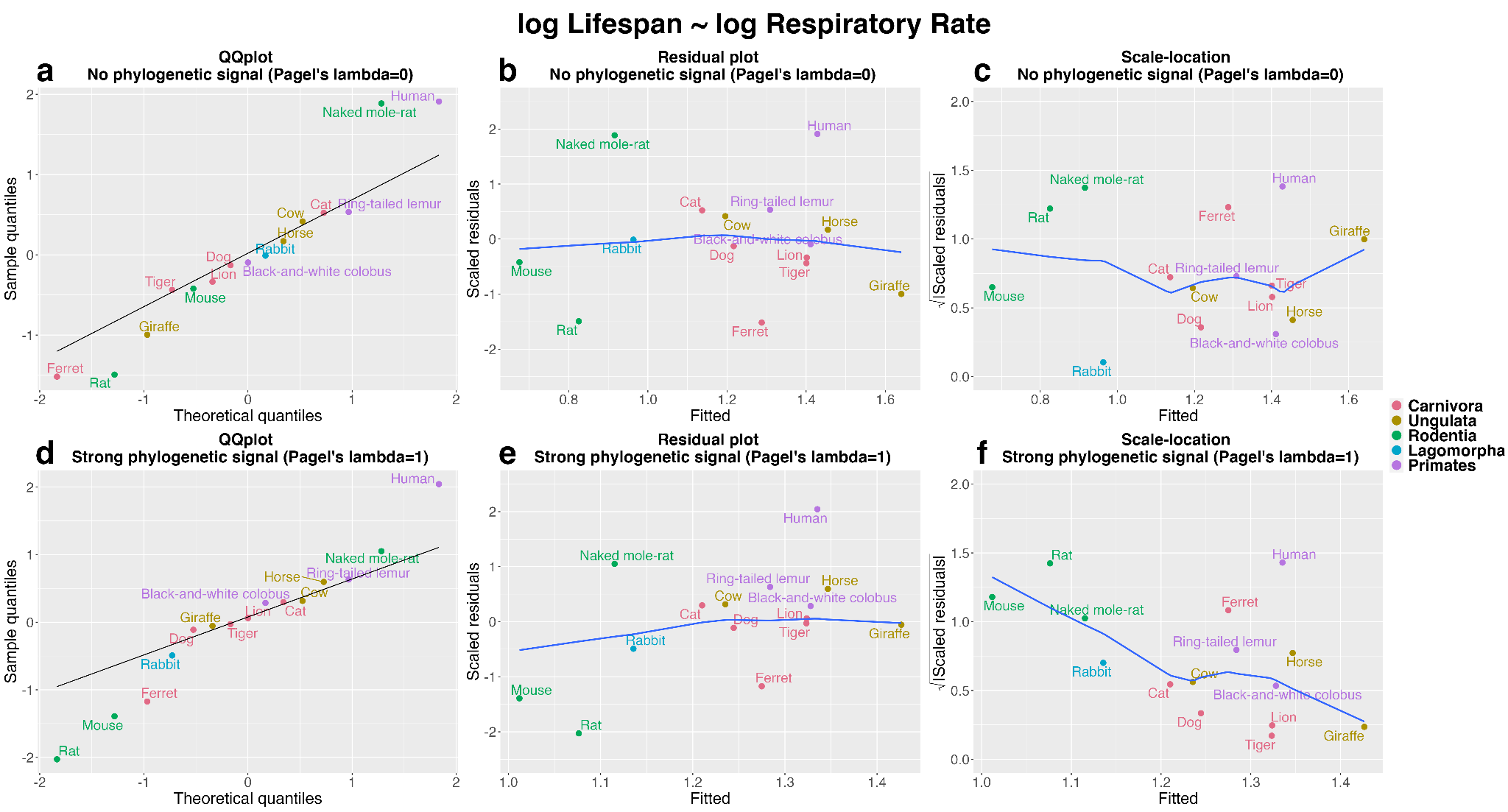
*

***Supplementary figure 2: Log lifespan as a function of log respiratory rate.*** *Here is shown the (****a****) QQplot, (****b****) residual and (****c****) scale-location plot of the univariate ordinary least squares (OLS) model. This model leverages lifespan as a function of respiratory rate, assuming statistical independence between the species (Pagel's lambda=0). In addition, we display the (****d****) QQplot,* *(****e****) residual and (****f****) scale-location plot of the univariate phylogenetic generalised least squares model (PGLS) model. This model uses lifespan as a function of respiratory rate, assuming a Brownian-motion model of evolution and a strong phylogenetic signal (Pagel's lambda=1).*

*
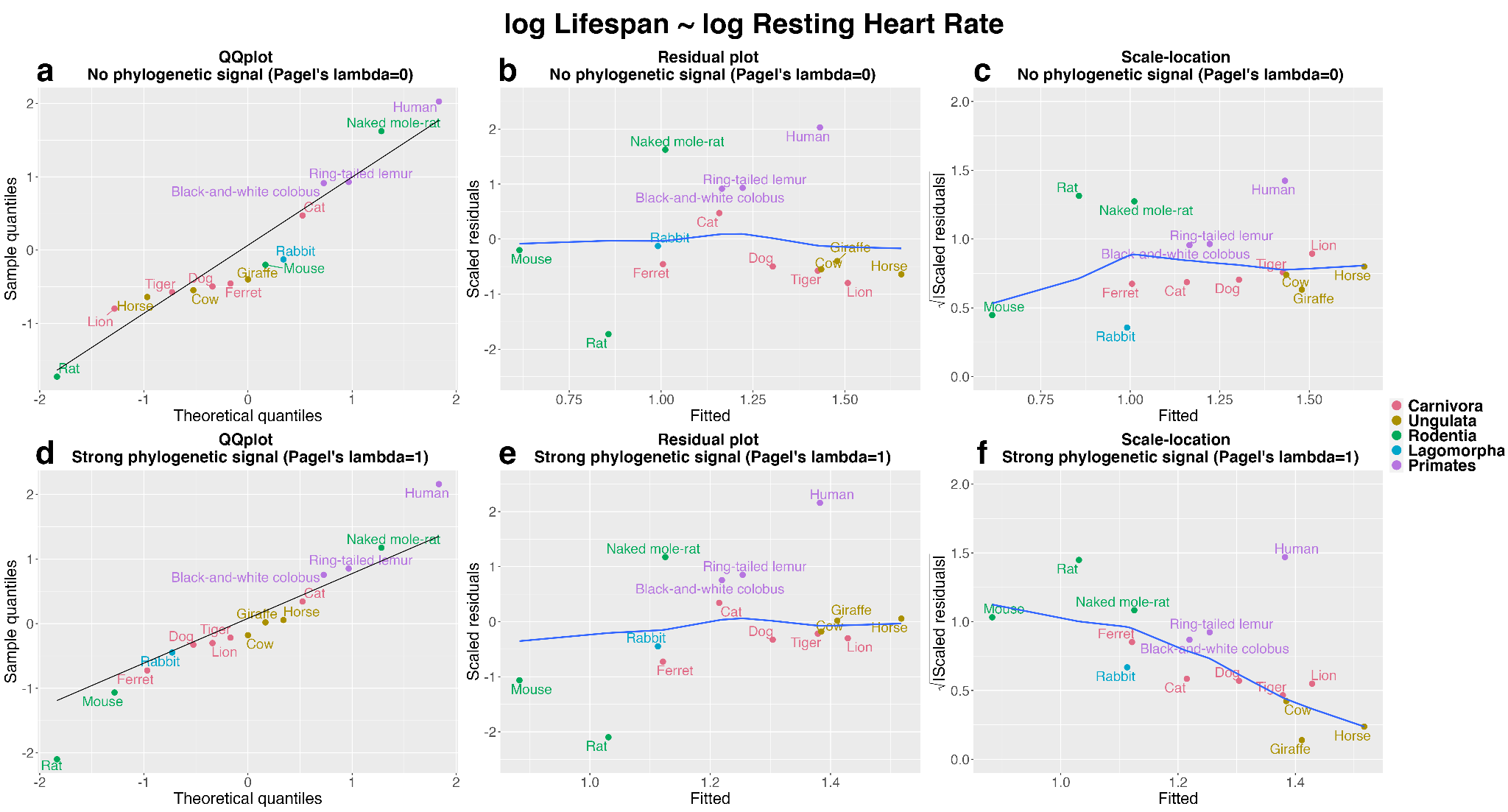
*

***Supplementary figure 3: Log lifespan as a function of log resting heart rate.*** *Here is shown the (****a****) QQplot, (****b****) residual and (****c****) scale-location plot of the univariate ordinary least squares (OLS) model. This model leverages lifespan as a function of resting heart rate, assuming statistical independence between the species (Pagel's lambda=0). In addition, we display the (****d****) QQplot,* *(****e****) residual and (****f****) scale-location plot of the univariate phylogenetic generalised least squares model (PGLS) model. This model uses lifespan as a function of resting heart rate, assuming a Brownian-motion model of evolution and a strong phylogenetic signal (Pagel's lambda=1).*

*
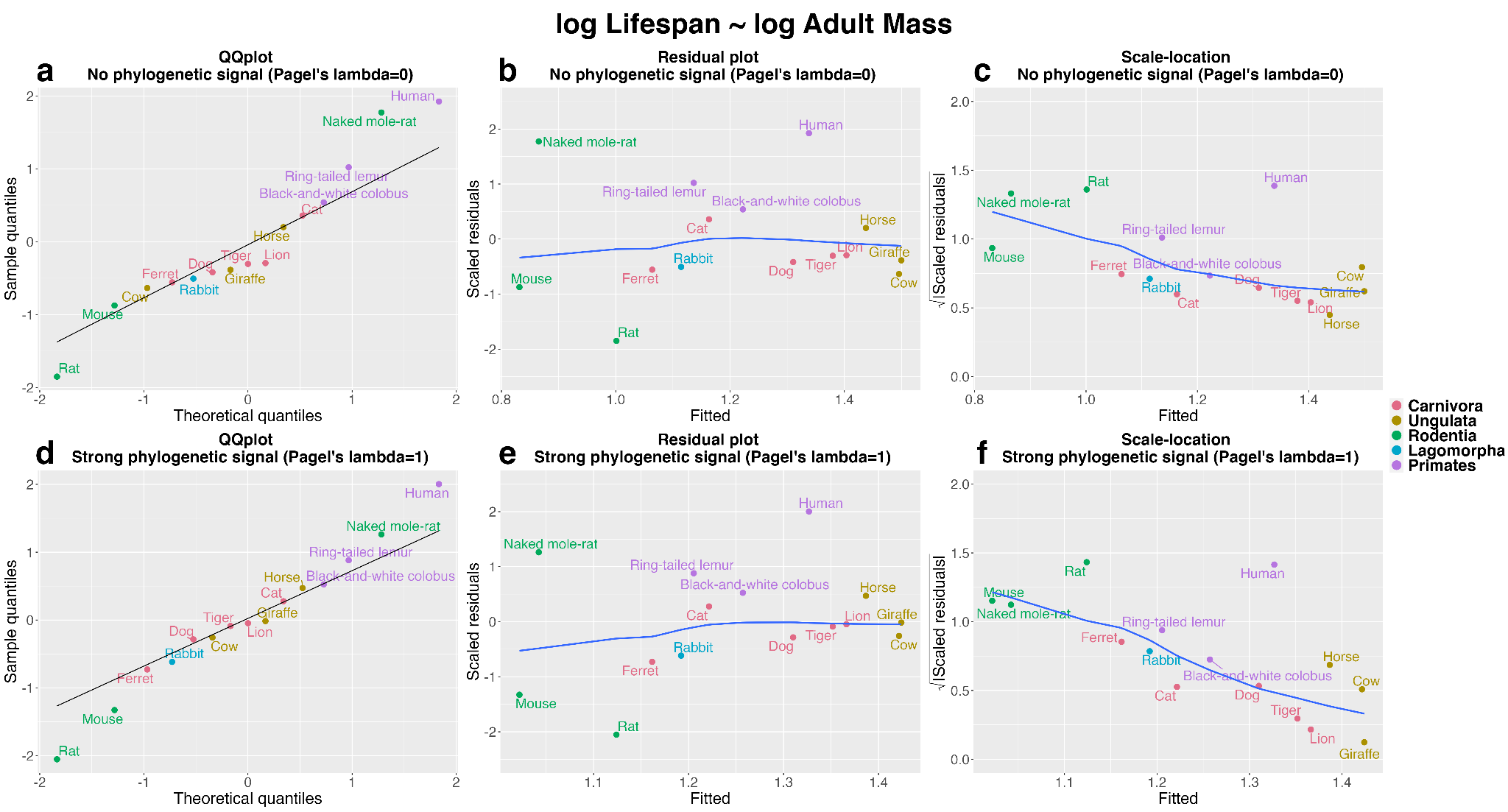
*

***Supplementary figure 4: Log lifespan as a function of log adult mass.*** *Here is shown the (****a****) QQplot, (****b****) residual and (****c****) scale-location plot of the univariate ordinary least squares (OLS) model. This model leverages lifespan as a function of adult mass, assuming statistical independence between the species (Pagel's lambda=0). In addition, we display the (****d****) QQplot,* *(****e****) residual and (****f****) scale-location plot of the univariate phylogenetic generalised least squares model (PGLS) model. This model uses lifespan as a function of adult mass, assuming a Brownian-motion model of evolution and a strong phylogenetic signal (Pagel's lambda=1).*

*
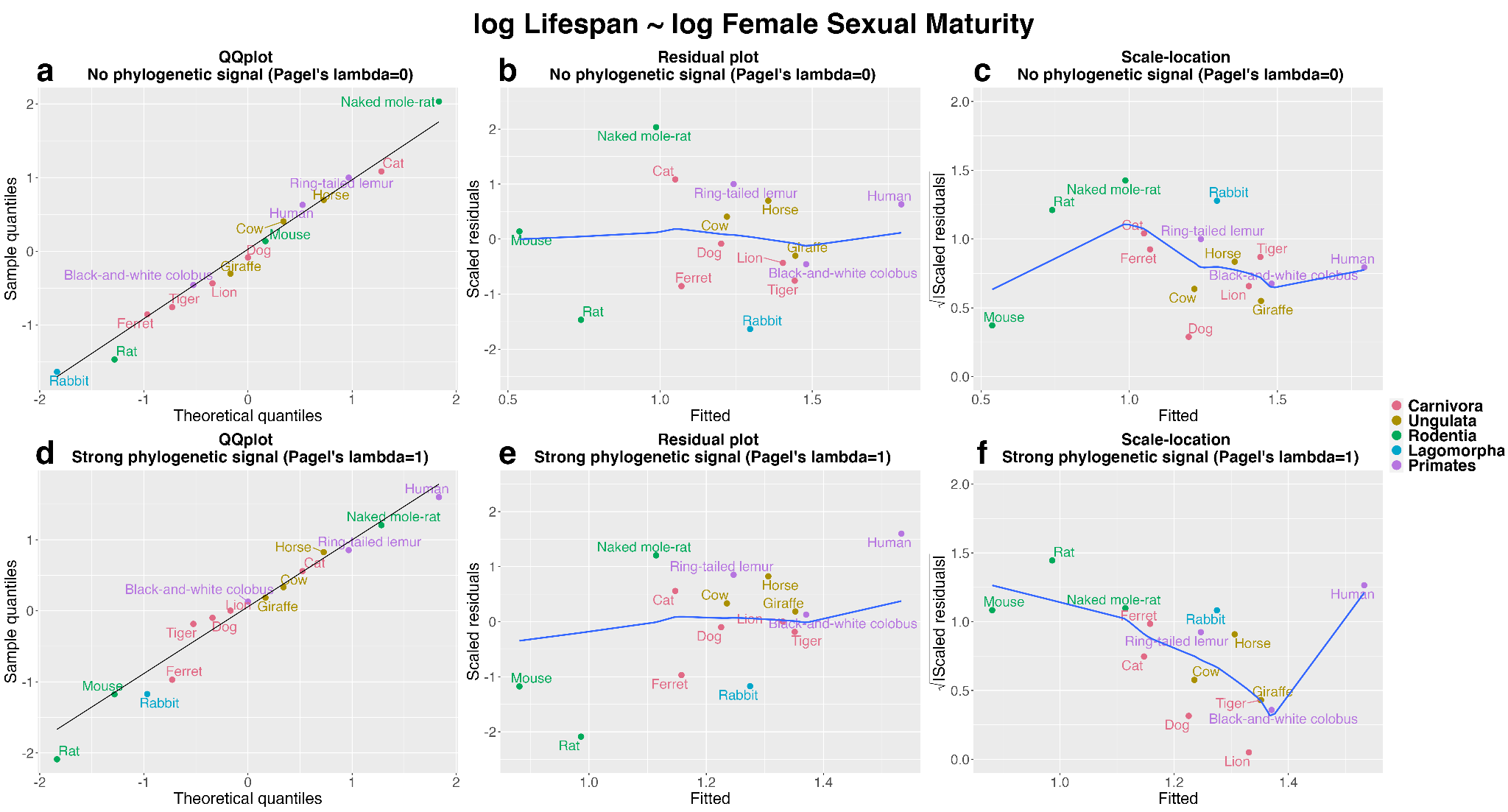
*

***Supplementary figure 5: Log lifespan as a function of log female sexual maturity.*** *Here is shown the (****a****) QQplot, (****b****) residual and (****c****) scale-location plot of the univariate ordinary least squares (OLS) model. This model leverages lifespan as a function of female sexual maturity, assuming statistical independence between the species (Pagel's lambda=0). In addition, we display the (****d****) QQplot,* *(****e****) residual and (****f****) scale-location plot of the univariate phylogenetic generalised least squares model (PGLS) model. This model uses lifespan as a function of female sexual maturity, assuming a Brownian-motion model of evolution and a strong phylogenetic signal (Pagel's lambda=1).*

*
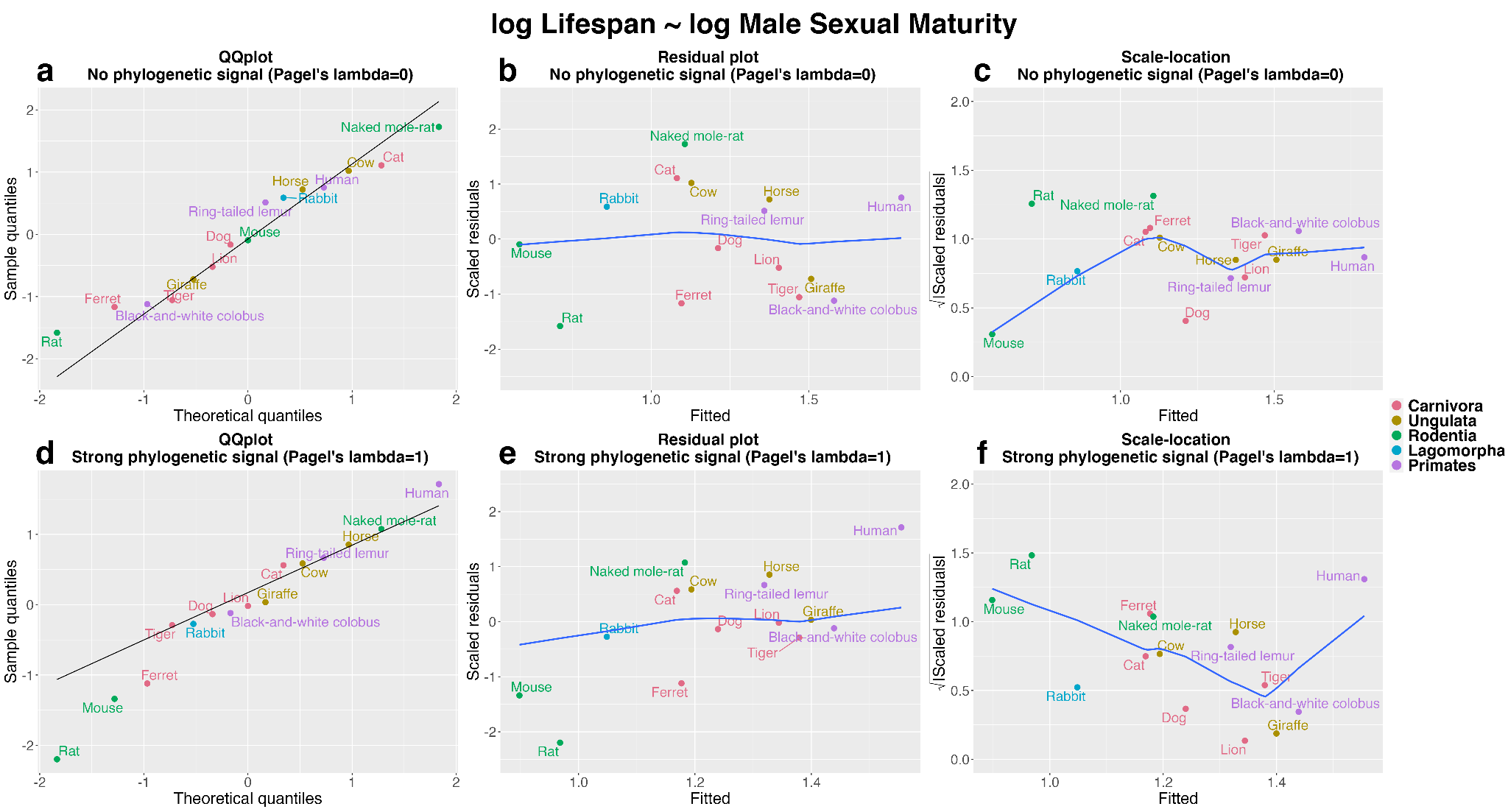
*

***Supplementary figure 6: Log lifespan as a function of log male sexual maturity.*** *Here is shown the (****a****) QQplot, (****b****) residual and (****c****) scale-location plot of the univariate ordinary least squares (OLS) model. This model leverages lifespan as a function of male sexual maturity, assuming statistical independence between the species (Pagel's lambda=0). In addition, we display the (****d****) QQplot,* *(****e****) residual and (****f****) scale-location plot of the univariate phylogenetic generalised least squares model (PGLS) model. This model uses lifespan as a function of male sexual maturity, assuming a Brownian-motion model of evolution and a strong phylogenetic signal (Pagel's lambda=1).*

*
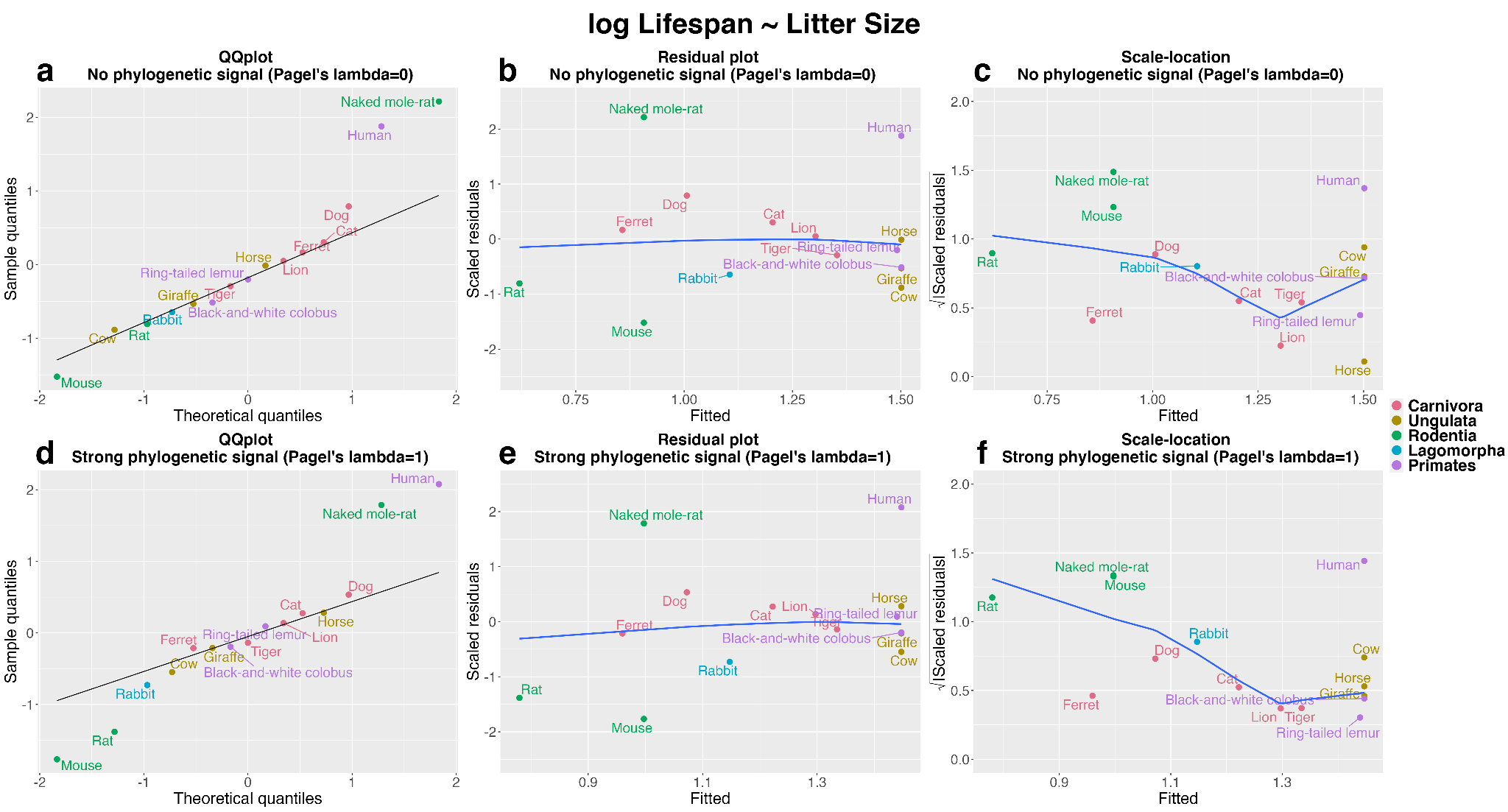
*

***Supplementary figure 7: Log lifespan as a function of litter size.*** *Here is shown the (****a****) QQplot, (****b****) residual and (****c****) scale-location plot of the univariate ordinary least squares (OLS) model. This model leverages lifespan as a function of litter size, assuming statistical independence between the species (Pagel's lambda=0). In addition, we display the (****d****) QQplot,* *(****e****) residual and (****f****) scale-location plot of the univariate phylogenetic generalised least squares model (PGLS) model. This model uses lifespan as a function of litter size, assuming a Brownian-motion model of evolution and a strong phylogenetic signal (Pagel's lambda=1).*

*
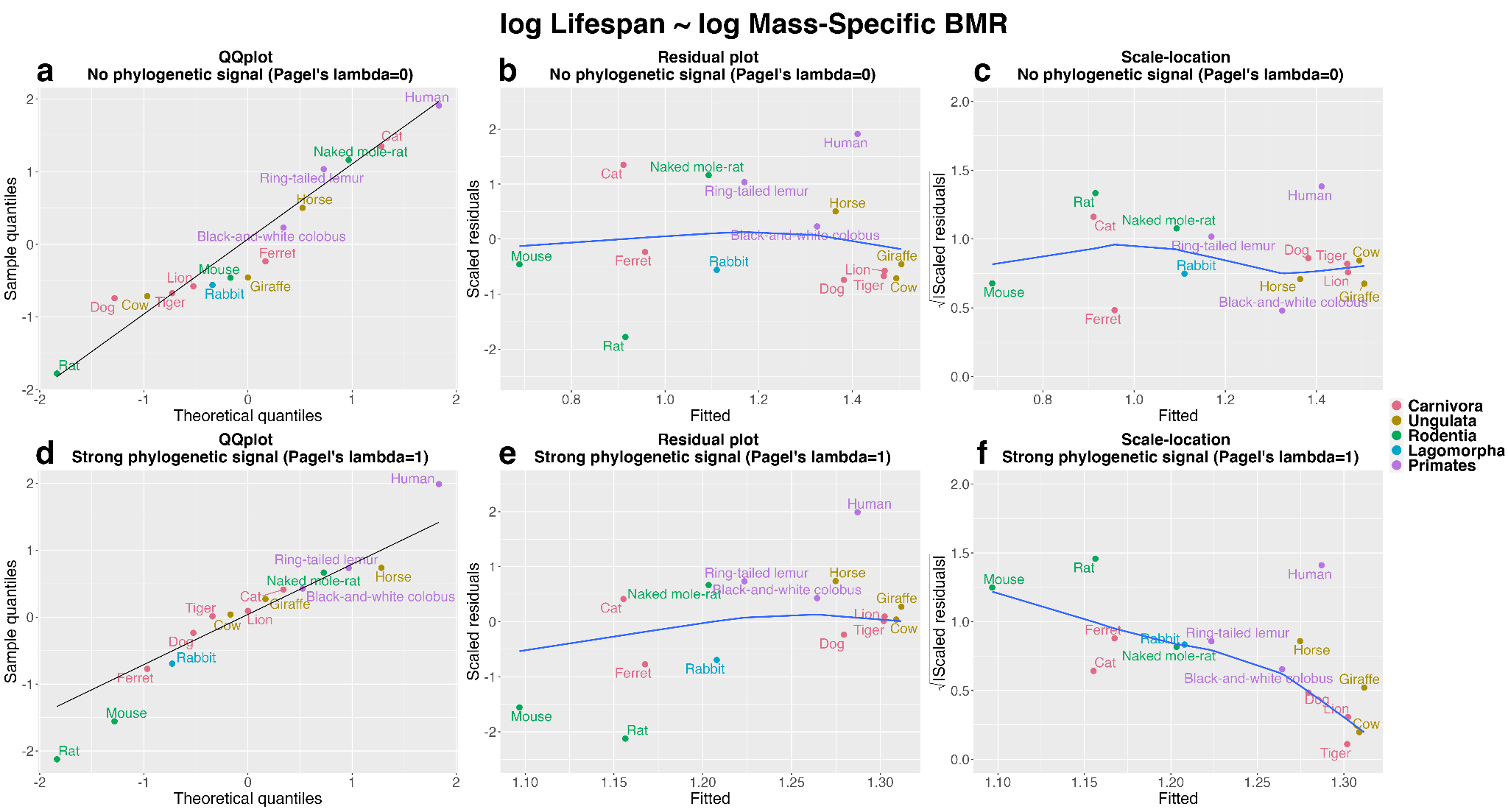
*

***Supplementary figure 8: Log lifespan as a function of log mass-specific basal metabolic rate (BMR).*** *Here is shown the (****a****) QQplot, (****b****) residual and (****c****) scale-location plot of the univariate ordinary least squares (OLS) model. This model leverages lifespan as a function of mass-specific basal metabolic rate, assuming statistical independence between the species (Pagel's lambda=0). In addition, we display the (****d****) QQplot,* *(****e****) residual and (****f****) scale-location plot of the univariate phylogenetic generalised least squares model (PGLS) model. This model uses lifespan as a function of mass-specific basal metabolic rate, assuming a Brownian-motion model of evolution and a strong phylogenetic signal (Pagel's lambda=1).*

*
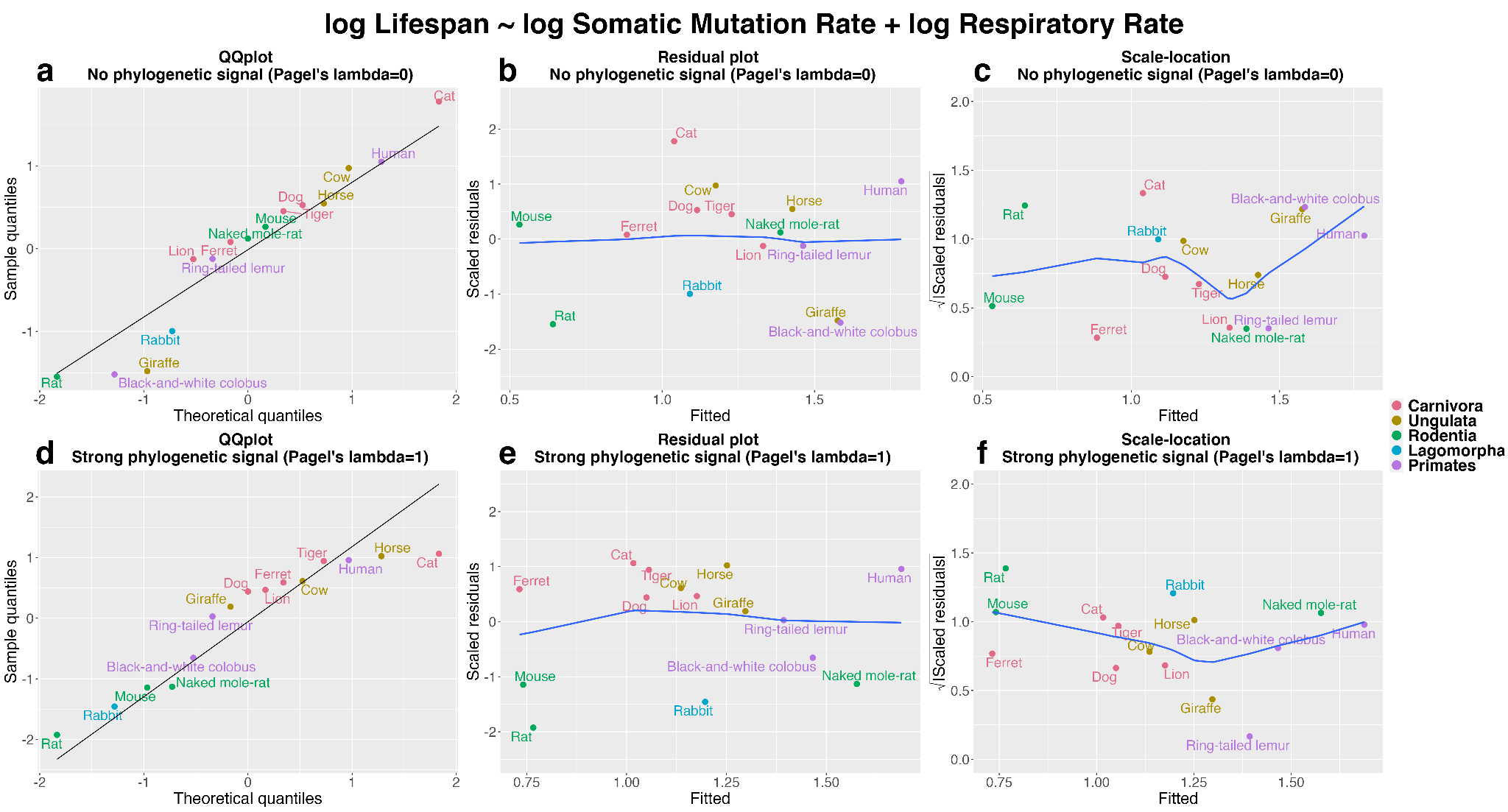
*

***Supplementary figure 9: Log lifespan as a function of log somatic mutation rate and log respiratory rate.*** *Here is shown the (****a****) QQplot, (****b****) residual and (****c****) scale-location plot of the multivariate ordinary least squares (OLS) model. This model leverages lifespan as a function of somatic mutation rate and respiratory rate, assuming statistical independence between the species (Pagel's lambda=0). In addition, we display the (****d****) QQplot,* *(****e****) residual and (****f****) scale-location plot of the multivariate phylogenetic generalised least squares model (PGLS) model. This model uses lifespan as a function of somatic mutation rate and respiratory rate, assuming a Brownian-motion model of evolution and a strong phylogenetic signal (Pagel's lambda=1).*

*
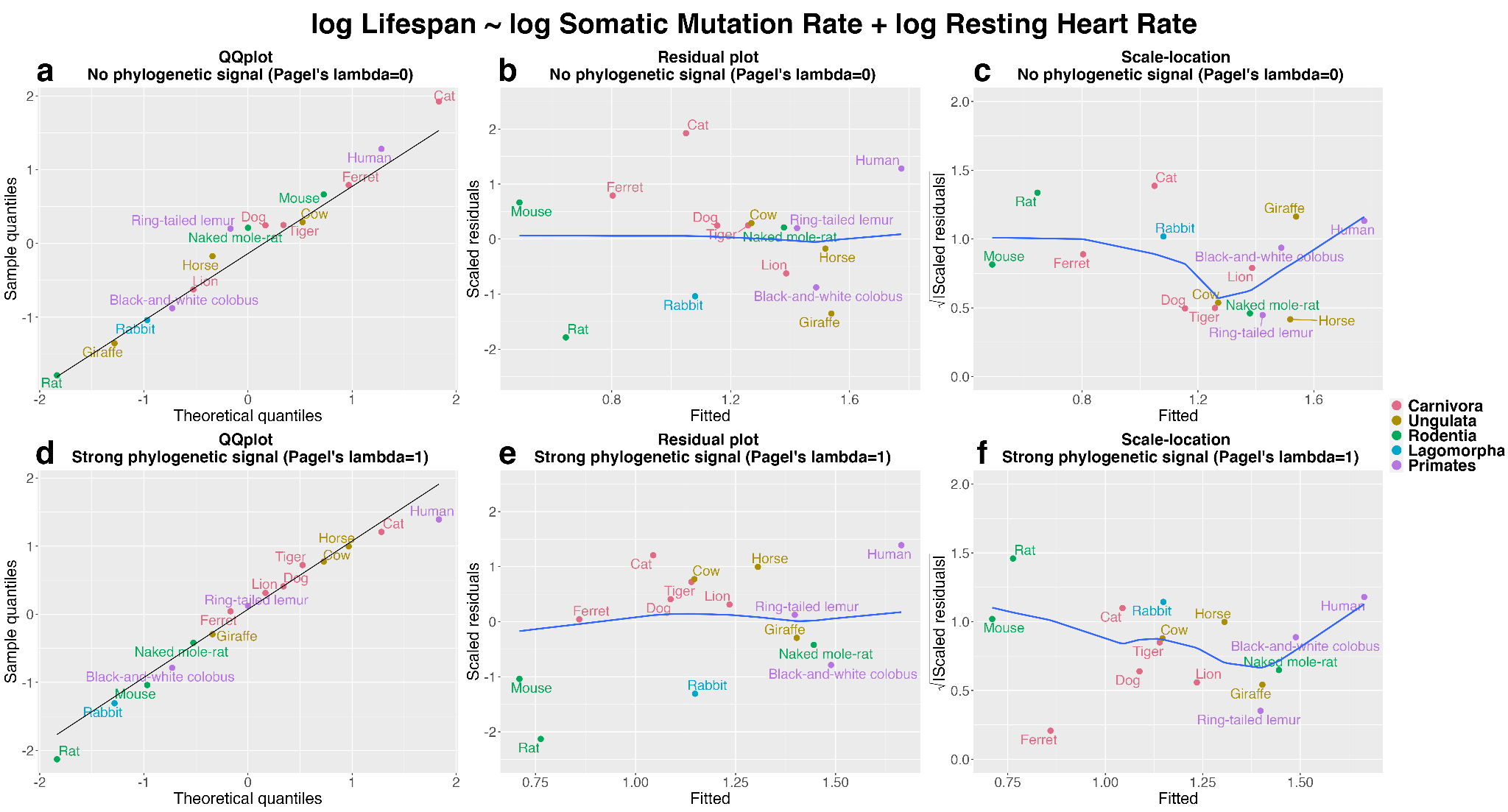
*

***Supplementary figure 10: Log lifespan as a function of log somatic mutation rate and log resting heart rate.*** *Here is shown the (****a****) QQplot, (****b****) residual and (****c****) scale-location plot of the multivariate ordinary least squares (OLS) model. This model leverages lifespan as a function of somatic mutation rate and resting heart rate, assuming statistical independence between the species (Pagel's lambda=0). In addition, we display the (****d****) QQplot,* *(****e****) residual and (****f****) scale-location plot of the multivariate phylogenetic generalised least squares model (PGLS) model. This model uses lifespan as a function of somatic mutation rate and resting heart rate, assuming a Brownian-motion model of evolution and a strong phylogenetic signal (Pagel's lambda=1).*

*
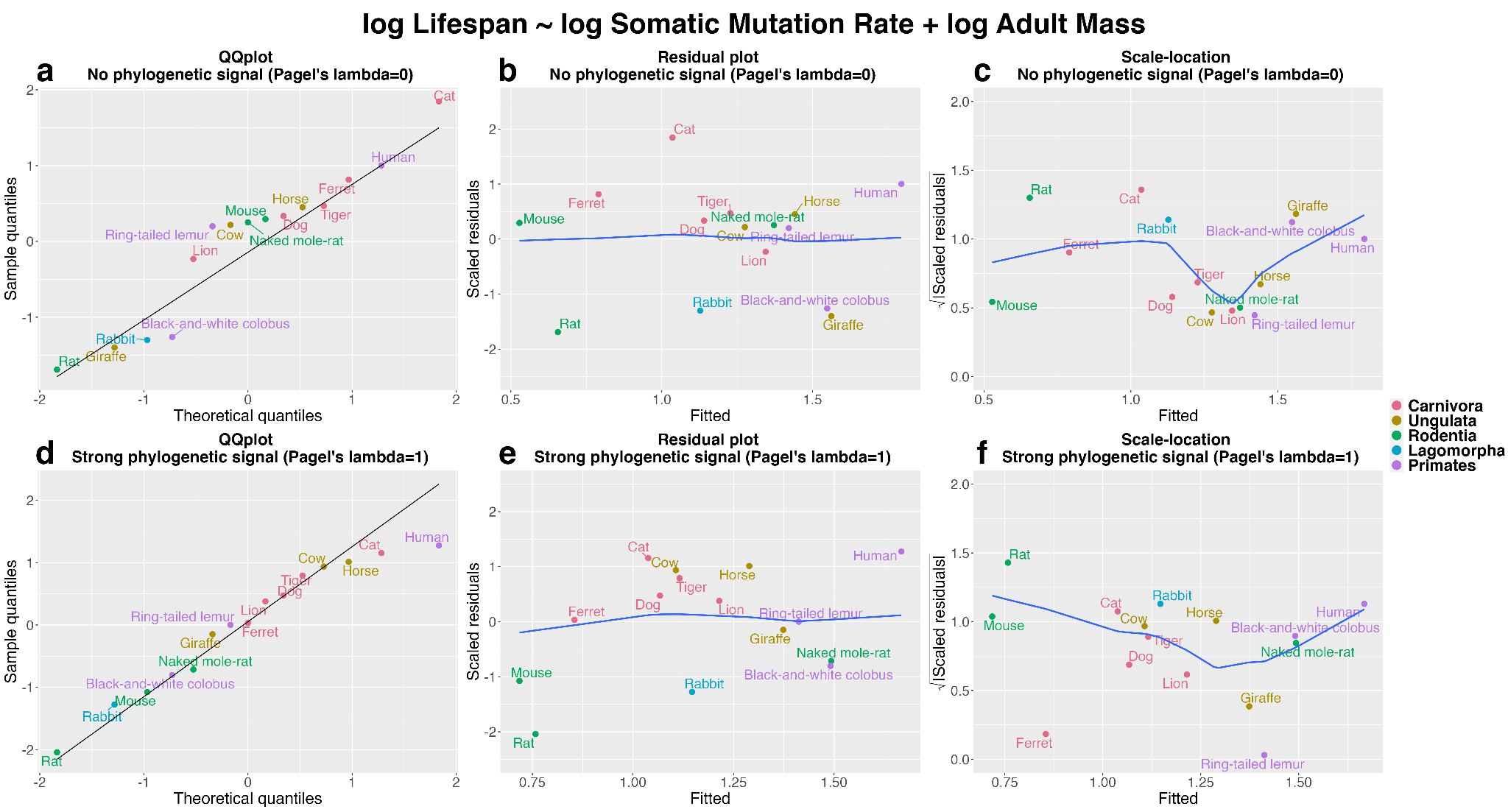
*

***Supplementary figure 11: Log lifespan as a function of log somatic mutation rate and log adult mass.*** *Here is shown the (****a****) QQplot, (****b****) residual and (****c****) scale-location plot of the multivariate ordinary least squares (OLS) model. This model leverages lifespan as a function of somatic mutation rate and adult mass, assuming statistical independence between the species (Pagel's lambda=0). In addition, we display the (****d****) QQplot,* *(****e****) residual and (****f****) scale-location plot of the multivariate phylogenetic generalised least squares model (PGLS) model. This model uses lifespan as a function of somatic mutation rate and adult mass, assuming a Brownian-motion model of evolution and a strong phylogenetic signal (Pagel's lambda=1).*

*
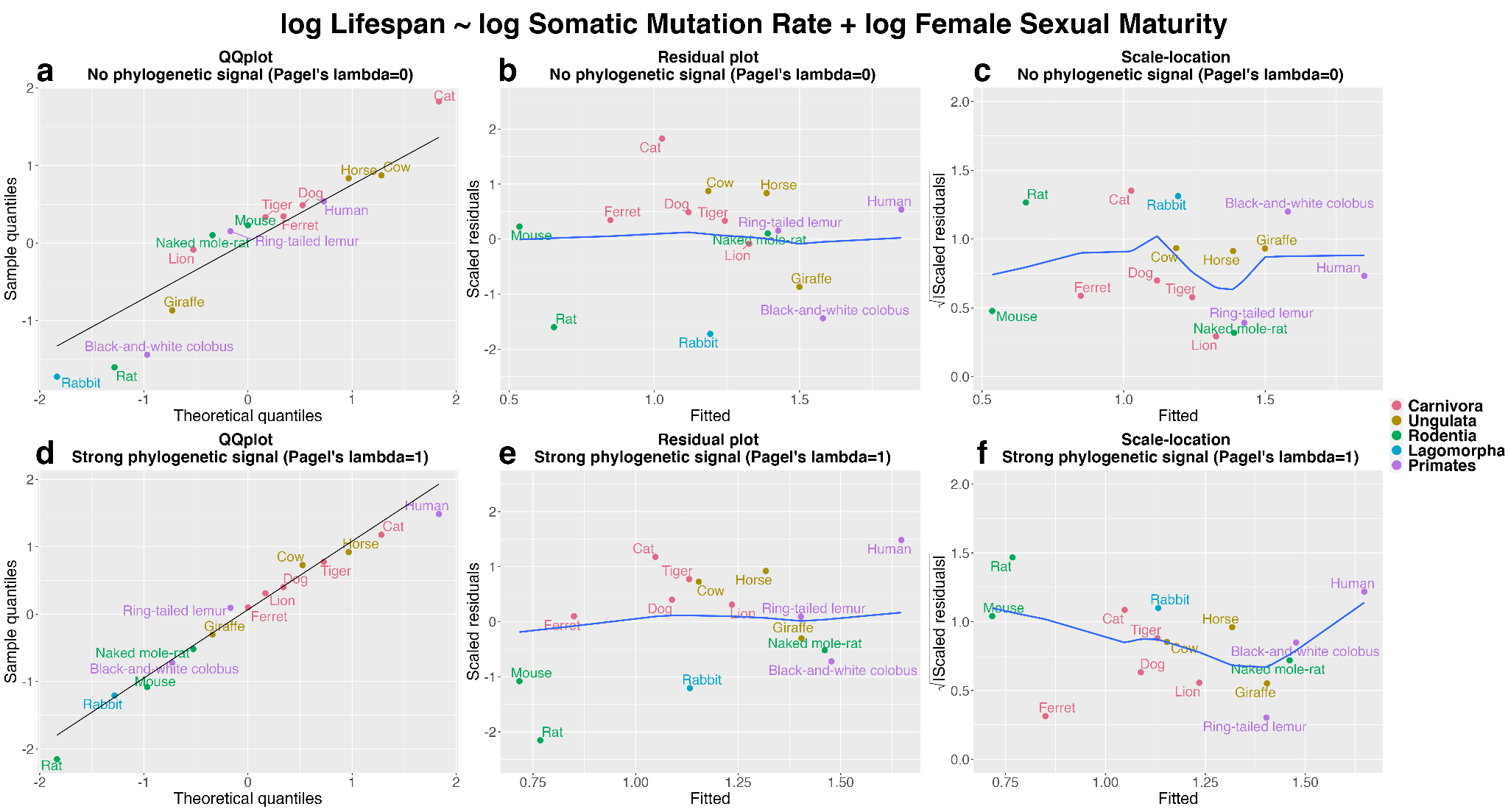
*

***Supplementary figure 12: Log lifespan as a function of log somatic mutation rate and log female sexual maturity.*** *Here is shown the (****a****) QQplot, (****b****) residual and (****c****) scale-location plot of the multivariate ordinary least squares (OLS) model. This model leverages lifespan as a function of somatic mutation rate and female sexual maturity, assuming statistical independence between the species (Pagel's lambda=0). In addition, we display the (****d****) QQplot,* *(****e****) residual and (****f****) scale-location plot of the multivariate phylogenetic generalised least squares model (PGLS) model. This model uses lifespan as a function of somatic mutation rate and female sexual maturity, assuming a Brownian-motion model of evolution and a strong phylogenetic signal (Pagel's lambda=1).*

*
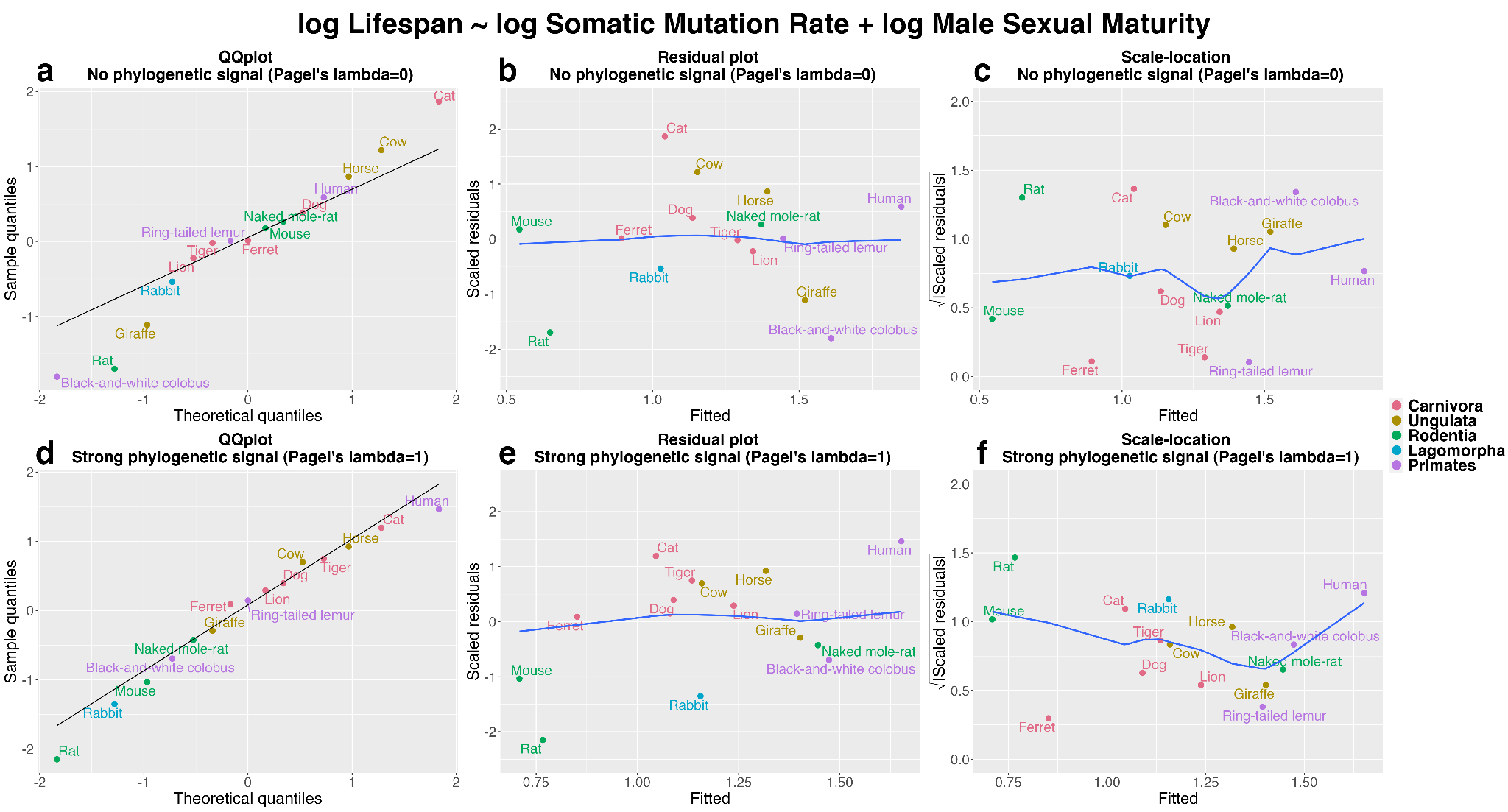
*

***Supplementary figure 13: Log lifespan as a function of log somatic mutation rate and log male sexual maturity.*** *Here is shown the (****a****) QQplot, (****b****) residual and (****c****) scale-location plot of the multivariate ordinary least squares (OLS) model. This model leverages lifespan as a function of somatic mutation rate and male sexual maturity, assuming statistical independence between the species (Pagel's lambda=0). In addition, we display the (****d****) QQplot,* *(****e****) residual and (****f****) scale-location plot of the multivariate phylogenetic generalised least squares model (PGLS) model. This model uses lifespan as a function of somatic mutation rate and male sexual maturity, assuming a Brownian-motion model of evolution and a strong phylogenetic signal (Pagel's lambda=1).*

*
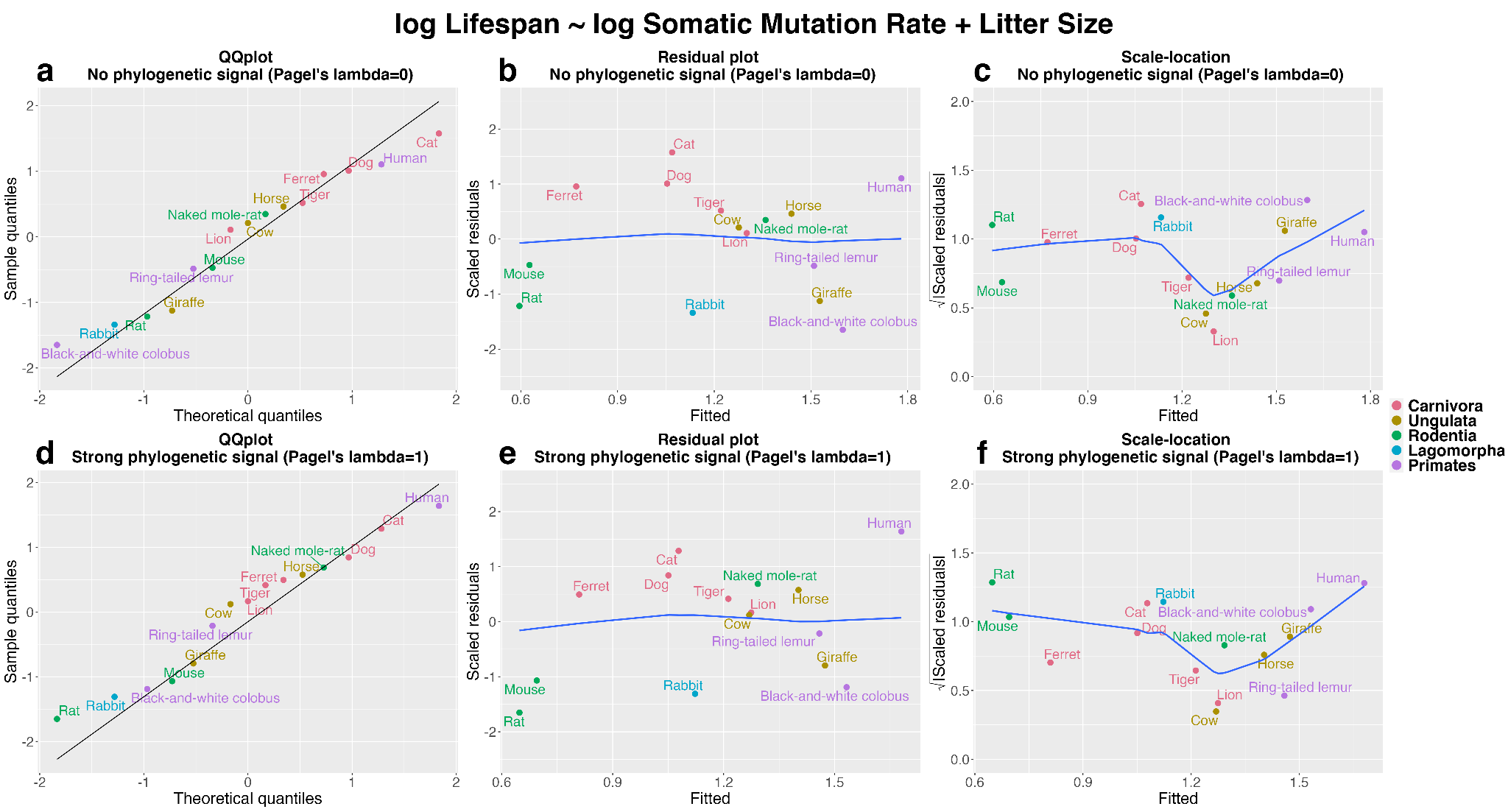
*

***Supplementary figure 14: Log lifespan as a function of log somatic mutation rate and litter size.*** *Here is shown the (****a****) QQplot, (****b****) residual and (****c****) scale-location plot of the multivariate ordinary least squares (OLS) model. This model leverages lifespan as a function of somatic mutation rate and litter size, assuming statistical independence between the species (Pagel's lambda=0). In addition, we display the (****d****) QQplot,* *(****e****) residual and (****f****) scale-location plot of the multivariate phylogenetic generalised least squares model (PGLS) model. This model uses lifespan as a function of somatic mutation rate and litter size, assuming a Brownian-motion model of evolution and a strong phylogenetic signal (Pagel's lambda=1).*

*
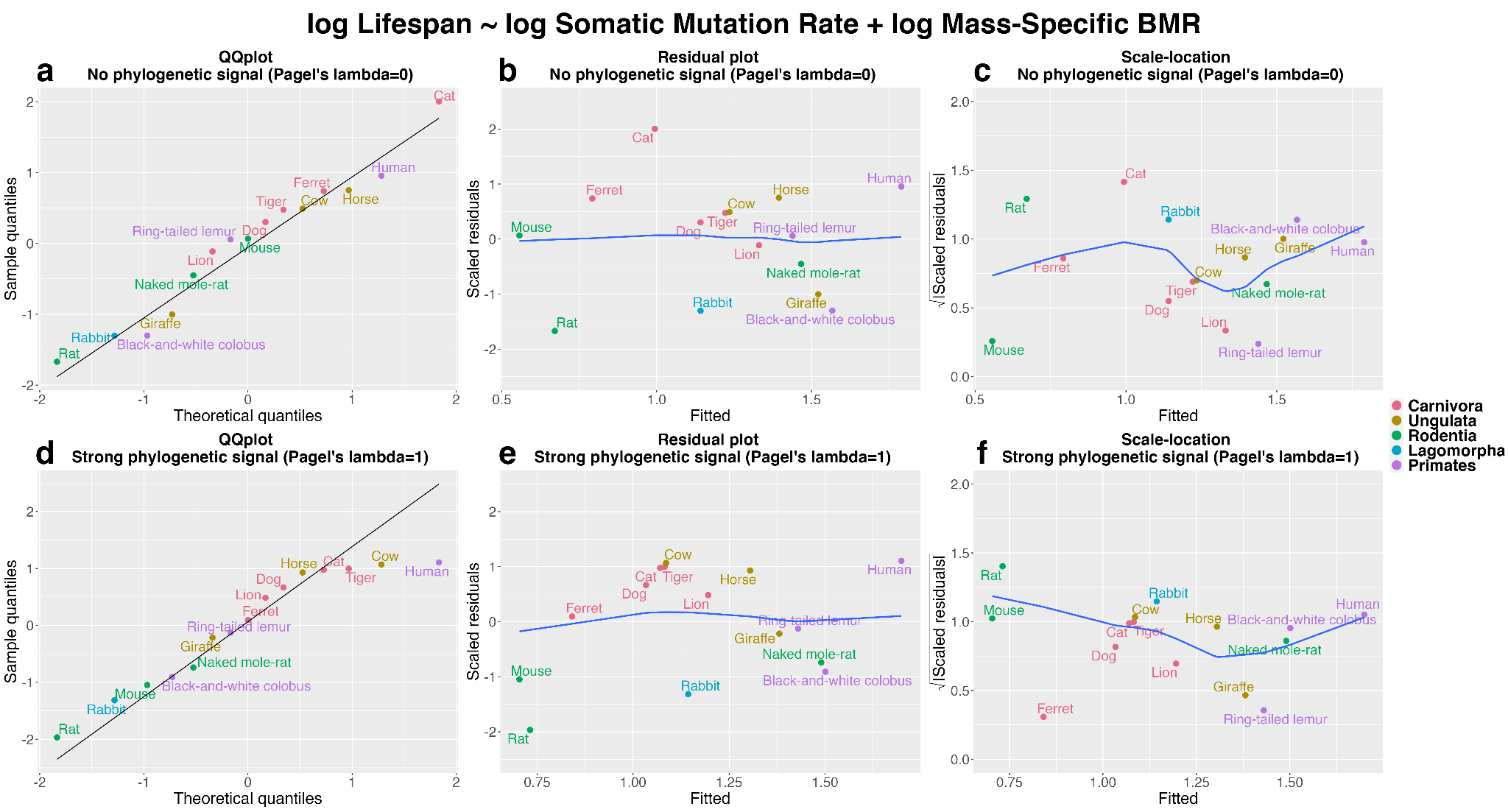
*

***Supplementary figure 15: Log lifespan as a function of log somatic mutation rate and log mass-specific basal metabolic rate (BMR).*** *Here is shown the (****a****) QQplot, (****b****) residual and (****c****) scale-location plot of the multivariate ordinary least squares (OLS) model. This model leverages lifespan as a function of somatic mutation rate and mass-specific basal metabolic rate, assuming statistical independence between the species (Pagel's lambda=0). In addition, we display the (****d****) QQplot,* *(****e****) residual and (****f****) scale-location plot of the multivariate phylogenetic generalised least squares model (PGLS) model. This model uses lifespan as a function of somatic mutation rate and mass-specific basal metabolic rate, assuming a Brownian-motion model of evolution and a strong phylogenetic signal (Pagel's lambda=1).*

***Supplementary figure 16: Distribution of the adjusted R-squared values of two OLS models predicting lifespan, bootstrapped dataset.*** *Here the distribution of the adjusted R-squared values of the two OLS models (****log lifespan ~ log somatic mutation rate*** *vs.* ***log lifespan ~ log somatic mutation rate + log resting heart rate****) are shown. The adjusted R-squared values were calculated for each bootstrapped dataset, in total for n=1,000 resamples.*
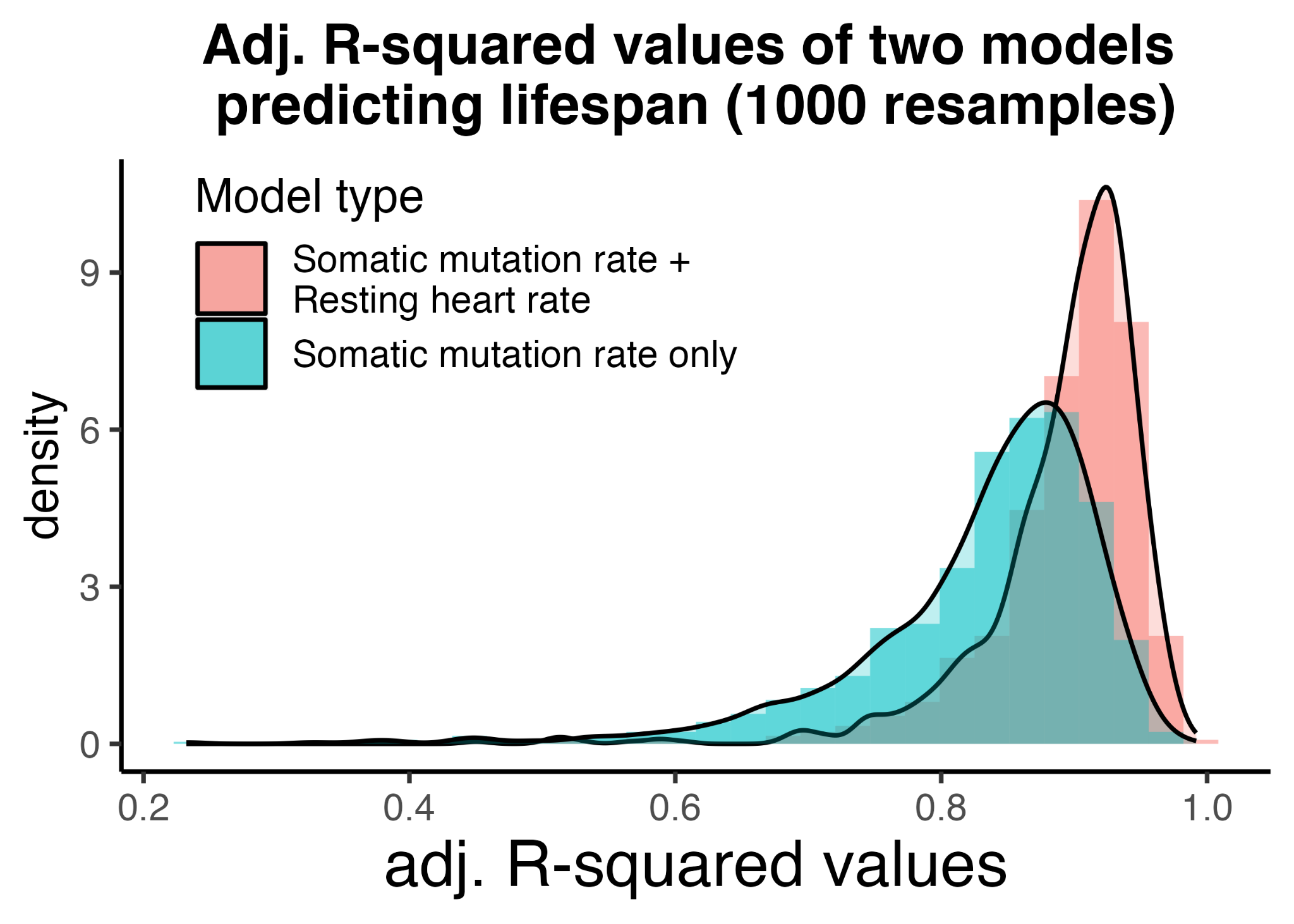


***
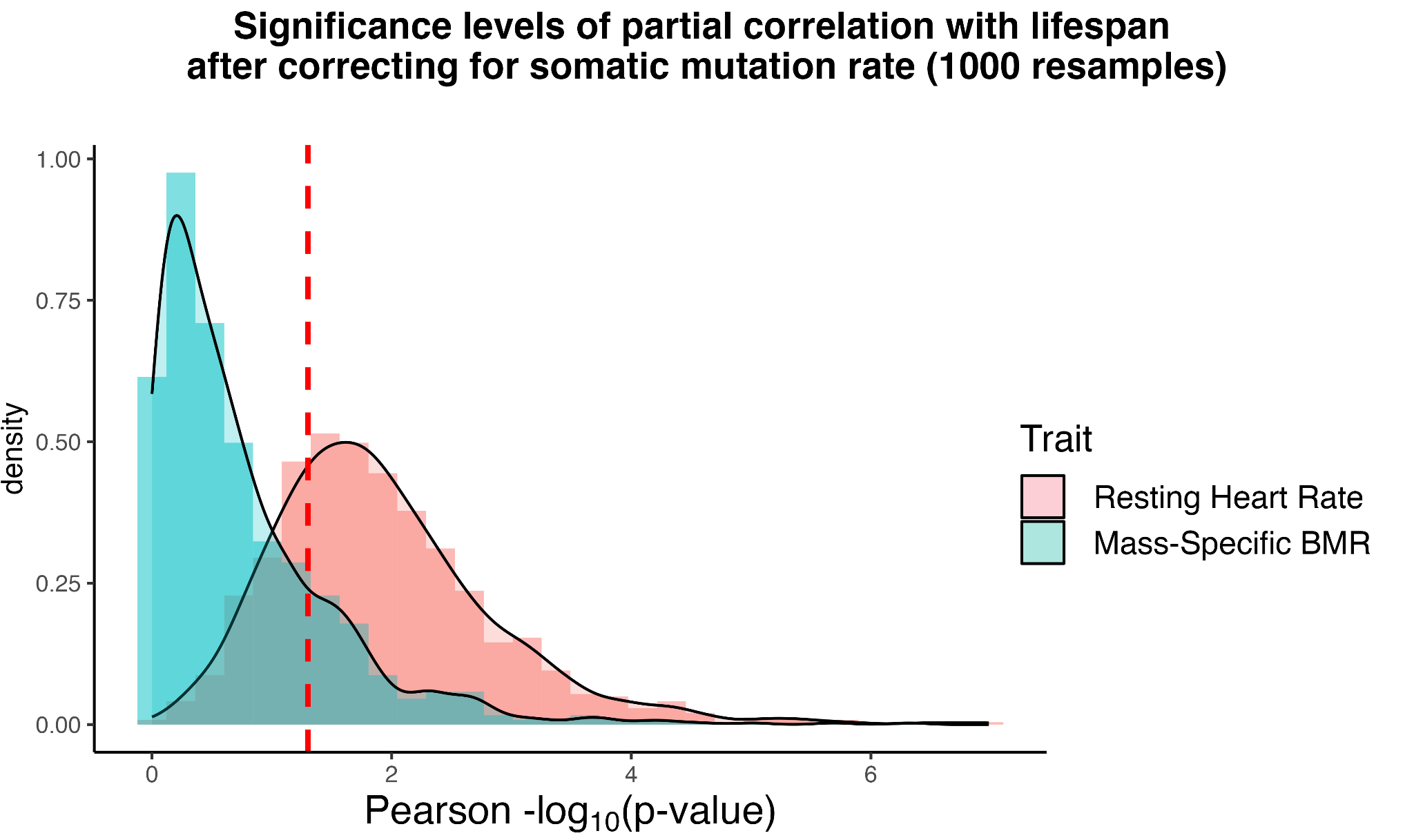
Supplementary figure 17: Distribution of significance levels of partial correlation with lifespan after correcting for somatic mutation rate, bootstrapped dataset .*** *For each bootstrapped dataset in the 1,000 resamples, we performed partial correlation analysis by controlling for somatic mutation rate’s effect, and calculated p-values of both resting heart rate’s and mass-specific basal metabolic rate’s (BMR) partial Pearson correlation with lifespan. Here shown is the distribution of the Pearson -log_10_  p-values for the bootstrapped dataset, the red dashed line indicates -log_10_(0.05) value.*
